# GluK1-containing kainate receptor-dependent long-term depression in the anterior cingulate cortex modulates chronic pain

**DOI:** 10.1186/s12967-026-08667-0

**Published:** 2026-07-17

**Authors:** Zhi-Xia Zhao, Ye Sun, Zi-Yuan Li, Jianxiong Wei, Teng Chen, Jingmin Che, Min Zhuo, Xu-Hui Li

**Affiliations:** 1 Frontier Institute of Science and Technology & College of Forensic Medicine, Xi’an Jiaotong University, Xi’an, Shaanxi 710061 China; 2 College of Forensic Medicine, Xi’an Jiaotong University Health Science Center, Xi’an, Shaanxi 710061 China; 3 Department of Physiology and Pathophysiology, School of Basic Medical Sciences, Xi’an Jiaotong University Health Science Center, Xi’an, Shaanxi 710061 China; 4 Shaanxi Provincial Key Laboratory of Infection and Immune Diseases, Shaanxi Provincial People’s Hospital, Xi’an, Shaanxi 710068 China; 5 Key Laboratory of Brain Aging and Neurodegenerative Diseases, Fujian Medical University, Fuzhou, China; 6 Department of Physiology, Faculty of Medicine, University of Toronto, Medical Science Building, 1 King’s College Circle, Toronto, ON Canada

**Keywords:** Kainate receptor, LTD, Chronic pain, Anterior cingulate cortex, ATPA

## Abstract

**Background:**

Long-term depression (LTD) is a pivotal form of synaptic plasticity in the central nervous system, yet its functions and cortical mechanisms remain unclear in chronic pain. Kainate receptors (KARs) are ionotropic glutamate receptors that regulate the release of both excitatory and inhibitory neurotransmitters in several brain areas, such as the anterior cingulate cortex (ACC), which is an important part of the brain for processing pain and emotions. KAR-dependent long-term potentiation (LTP) in the ACC has been proven to contribute to chronic pain. However, the existence of KAR-mediated LTD and its functional relevance to chronic pain remain unexplored.

**Methods:**

A multidisciplinary approach combining multi-channel field potential recording, whole-cell patch-clamp recording, and pharmacological assays was used to characterize KAR-mediated LTD in the ACC. The regulatory role of KAR-mediated LTD in chronic pain was evaluated using the spared nerve injury (SNI) mouse model, along with viral overexpression and knockdown techniques, as well as pharmacological administration of the KAR agonist ATPA.

**Results:**

Application of the KAR agonist kainic acid (KA) or the GluK1-selective agonist ATPA induced robust, dose-dependent LTD. The induction of LTD required the GluK1 subunit and L-type voltage-gated calcium channels but was independent of NMDA receptors. The Ca²⁺-activated PKA signaling pathway regulated this LTD, and presynaptic inhibition via GABA_A_ receptors was also involved. In spared nerve injury (SNI) mice, KAR-mediated LTD was occluded in the ACC. Knockdown of GluK1-containing KARs in excitatory neurons or their overexpression in inhibitory neurons produced analgesic effects in SNI mice. Moreover, intra-ACC microinjection of the GluK1 agonist ATPA attenuated mechanical and thermal hyperalgesia in both neuropathic and inflammatory pain models, without affecting anxiety-like responses.

**Conclusions:**

We identified a novel form of KAR‑mediated LTD in the ACC that depends on GluK1‑containing KARs and the Ca²⁺‑activated PKA signaling pathway. This form of LTD is involved in modulating chronic pain. These findings suggest that GluK1-containing KARs in the ACC represent a potential intervention node for chronic pain, warranting further preclinical investigation.

## Background

Long-term potentiation (LTP) and long-term depression (LTD) are two forms of synaptic plasticity in the mammalian central nervous system (CNS), extensively characterized in the context of learning and memory [1, 2]. Mounting evidence implicates that LTP and LTD are also involved in the processing of chronic pain, which can be thought of as a type of persistent sensory memory [3–7]. Clinical studies demonstrated that a long-term increase in perceived pain to electrical test stimuli was induced by high-frequency electrical stimulation (HFS) (5 × 1 s at 100 Hz) of peptidergic cutaneous afferents. In contrast, a long-term decrease in perceived pain was induced by low-frequency stimulation (LFS) (17 min at 1 Hz) [8]. Peripheral tissue injuries, such as digit or tail amputation, in rodents impair the induction of LTD in the anterior cingulate cortex (ACC), a key area for the integration of pain perception and emotional regulation [4, 9]. Wang YJ et al. (2020) also found that peripheral nerve injury inhibits LTD in the ACC and that the restoration of LTD in the ACC alleviates peripheral pain hypersensitivity [5]. Collectively, these findings indicate that loss of LTD is one of the key features of chronic pain.

Both ionotropic glutamate receptors (iGluRs) and metabotropic glutamate receptors (mGluRs) are involved in LTD induction. Two major forms of LTD have been characterized in the ACC: NMDAR-dependent and mGluR-dependent [1, 10, 11]. Kainate receptors (KARs), a subfamily of iGluRs, are critical modulators of synaptic transmission and plasticity in several brain regions, including the spinal cord, ACC, striatum, amygdala, and hippocampus [12–16]. Repetitive stimulation with 200 stimuli at 5 Hz (-70 mV holding potential) in acute brain slices generates KAR-mediated LTD in the perirhinal cortex and hippocampus, which is a mechanism postulated to underlie working memory [17, 18]. In addition, sustained chemical activation of KARs subunit GluK2 by kainic acid (KA) in hippocampal neurons has been shown to drive AMPAR endocytosis, thereby establishing LTD [19]. However, KAR-mediated LTD has not been reported in the ACC, whereas KARs mediating presynaptic LTP have been found in the ACC [20, 21].

In the ACC synapses, KARs have been reported to contribute to excitatory synaptic transmission and modulate presynaptic transmitter release [15, 22, 23]. The postsynaptic KARs contribute approximately 10–15% of fast excitatory synaptic transmission in the ACC neuron. Beyond this postsynaptic role, presynaptic KARs bidirectionally modulate both excitatory and inhibitory transmission. Specifically, the activation of KARs subunit GluK1 triggers action potential-dependent GABA release, a process contingent upon voltage-dependent calcium channel activation and subsequent Ca^2+^ influx [24, 25]. Recently, our group demonstrated that KAR-mediated excitatory transmission in the ACC is significantly increased in the tree shrew (a primate-like animal) compared to mice [23]. Moreover, they are involved in the regulation of synaptic transmission in the bilateral ACC cortico-cortical connections and upregulated in ACC-corticostriatal circuits related to seizure-like activity [14]. In chronic pain, KARs are thought to disturb the critical balance of excitation and inhibition (E/I) transmission [15, 26–28]. Despite extensive research on ACC KARs, the role of the KA receptor in the inhibition of synaptic transmission is not well understood. The presence of KAR-mediated LTD and its functional significance to chronic pain has not been investigated.

In the present study, we employed a multidisciplinary approach combining multi-channel field potential recording, whole-cell patch-clamp recording, pharmacology, and behavioral assays to characterize KAR-mediated LTD in the ACC. We demonstrate the existence of a novel KAR-mediated LTD in the ACC that is mediated by GluK1-containing KARs. Furthermore, pharmacological intervention with ATPA, specific knockdown of GluK1 in excitatory neurons, or overexpression of GluK1 in inhibitory neurons produced significant analgesia in a spared nerve injury (SNI) mouse model. Our results demonstrate that GluK1‑containing KAR‑mediated LTD in the ACC is involved in the maintenance of chronic pain. Furthermore, pharmacological activation of GluK1 or cell‑type‑specific modulation of GluK1 expression produces analgesic effects, supporting that GluK1‑containing KARs in the ACC may serve as a candidate intervention node for chronic pain.

## Methods

### Animals

The animals used were adult male C57BL/6 mice (6–8 weeks old), which were purchased from the Experimental Animal Center of Xi’an Jiaotong University. The adult male CaMKIIα-Cre and male GAD-Cre mice were purchased from Jaxlab. All mice were housed under a 12 h light/dark cycle (8:30 a.m. to 8:30 p.m. light) in a holding room kept at a temperature of 23 ± 1℃ with food and water provided ad libitum. The Animal Care and Use Committee of Xi’an Jiaotong University approved all experimental protocols.

### Drug application

Kainic acid (KA), KAR agonist; (RS)-2-amino-3-(3-hydroxy-5-tert-butylisoxazol-4-yl) propanoic acid (ATPA), GluK1-selective agonist; UBP310, GluK1-selective antagonist; D (-)-2-amino-5-phosphonopentanoic acid (AP-5), competitive NMDAR antagonist; Naspm, CP-AMPAR antagonist; Chelerythrine chloride, PKC inhibitor; KT-5720, PKA inhibitor; Nimodipine (L-VGCC blocker) and GYKI 53,655 hydrochloride (Non-competitive, selective AMPAR antagonist) were obtained from Tocris Cookson (Bristol, UK). Picrotoxin (GABA_A_ receptor antagonist) was obtained from Sigma Aldrich (Canada). Kainic acid (1, 5, 10 µM), ATPA (1, 10, 30 µM), AP-5 (50, 100 µM), and Naspm (50 µM) were dissolved in distilled water. UBP310 (20 µM), Chelerythrine chloride (3 µM), KT-5720 (2 µM), Nimodipine (20 µM), and GYKI 53,655 hydrochloride (100 µM) were dissolved in dimethyl sulfoxide (DMSO). Picrotoxin (100 µM) was dissolved in ethanol. Drugs were prepared as stock solutions for frozen aliquots at -20℃. All the drugs were diluted from the stock solutions to the final desired concentration in the ACSF before being applied to the perfusion solution. The DMSO diluted in ACSF did not affect basal synaptic transmission and plasticity.

### Chronic neuropathic pain model

#### Spared nerve injury (SNI) mouse model

The spared nerve injury (SNI) mouse model was used to induce chronic neuropathic pain [14]. Briefly, mice were anesthetized by 2% isoflurane. An incision was made on the skin and muscle of the left thigh to expose three branches of the sciatic nerve. The common peroneal nerve (CPN) and tibial nerve (TN) were tightly ligated with 5 − 0 silk suture, and the nerve segments were transected distal to the ligation sites (away from the spinal cord). Leave the sural nerve (SN) intact. Then, the muscle and skin were sutured. The sham operation included exposing the sciatic nerve but not damaging any nerves. All animals were kept in a temperature-controlled recovery chamber for at least 30 min after surgery.

#### CFA-induced inflammatory pain mouse model

A homogeneous emulsion was prepared by mixing Complete Freund’s Adjuvant (CFA; Sigma-Aldrich, USA) with 0.9% sterile saline at a 1:1 volume ratio using a vortex oscillator [29]. A microsyringe was employed to accurately aspirate 10 µL of the 50% CFA emulsion, which was then injected subcutaneously into the plantar surface of the left hind paw. The needle was retained for 20 s post-injection to ensure proper deposition of the emulsion, then slowly withdrawn to prevent leakage.

### Cannula implantation and microinjection of drugs

Cannulation and microinjection were performed as described previously [30]. After anesthetizing mice with 2% isoflurane, they were fixed to the stereotaxic device, and an incision was made over the skull to expose the surface. The cross line was exposed, starting from the bregma, and two small holes were drilled + 0.9 mm forward and ± 0.4 mm on both sides of the midline. Then the cannula was buried 1.5 mm on the ventral side of the bilateral craniofacial surface and fixed with dental cement. Microinjection and behavioral tests were performed 14 days after surgery. Microinjection was performed using an injection pump (ALC-IP800, Alcbio, Shanghai, China) and a Hamilton syringe (Hamilton, Reno, NY, USA).

Bilateral injections of 0.5 µL/side over 1 min into the ACC (Total 1 µL for each mouse). The volume delivered was confirmed by observing the downward movement of the meniscus in calibrated polyethylene (PE10) tubing. The injector was left in place for an additional 1 min after injection to minimize drug reflux. ATPA (0.25, 1.25, and 3.75 ng) or sterile saline as a vehicle was delivered bilaterally into the ACC of mice (25 g). After microinjection, the mice were tested for their mechanical pain threshold and thermal pain threshold immediately. After the behavioral tests, we confirmed the injection sites histologically.

### Virus injection

AAV2/9-DIO-mCherry-miR30shRNA(*Grik1*)-WPRE (1.70 × 10^12^ vg/mL; the shRNA target sequence against GluK1: 5′-CCTGGACATTATCAGTCTCA-3′), AAV2/9-DIO-mCherry-miR30shRNA(NC)-WPRE (1.10 × 10^12^ vg/mL), or AAV2/9-DIO-*Grik1*-3×FLAG-tWPA (1.80 × 10^12^ vg/mL; *Grik1* Gene ID:14805, Transcript: NM_146072.5, full-length coding sequence 2877 bp), AAV2/9-DIO-mCherry-3×FLAG- tWPA (1.60 × 10^12^ vg/mL) were purchased from OBIO (Shanghai, China). Positioning and drilling were similar to cannula implantation. The viruses were injected with equal speed by a microsyringe pump (NANOJECT II, Drummond Scientific Company) into the ACC location in CaMKII-Cre adult mice or GAD-Cre adult mice. The injection volume and flow rate were 300 nL at 50 nL/min. After the injection, the surgical wound was carefully sutured and disinfected. The animals were monitored until they woke up, then returned to their home cages.

### Brain slices preparation

Coronal brain slices, including the ACC, were prepared using our previous methods [13, 30]. Briefly, adult male mice were anesthetized with isoflurane. The brains were quickly removed and transferred to ice-cold oxygenated (95% O_2_ and 5% CO_2_) artificial cerebrospinal fluid (ACSF) containing (in mM): 124 NaCl, 2.5 KCl, 2 CaCl_2_, 2 MgSO_4_, 25 NaHCO_3_, 1 NaH_2_PO_4_, and 10 glucose, pH 7.4. Then, the brain was glued to the cutting stage tissue slicer (Leica, VT1200S) for coronal ACC slices (300 μm thickness per slice). The slices were transferred to a submerged recovery chamber with oxygenated (95% O_2_ and 5% CO_2_) ACSF at room temperature for at least 1 h.

### Preparation of the multielectrode array

The multielectrode array system used in the current study was a MED64 recording system (P515A, Panasonic Alpha-Med Sciences). The MED64 probe has an array of 64 planar microelectrodes, arranged in an 8 × 8 pattern, with an interelectrode distance of 150 μm. Brain slices can be better attached to MED64 probes. The MED64 probe requires a hydrophilic treatment before use. The surface of the MED64 probe was treated with 0.1% polyethyleneimine (P-3143, Sigma-Aldrich) in 25 mmol/L borate buffer, pH 8.4, overnight at room temperature. Then, the probe surface was rinsed at least three times with sterile distilled water.

### Multichannel field potential recording in the ACC slices of adult male mice

**Fig. 1:**
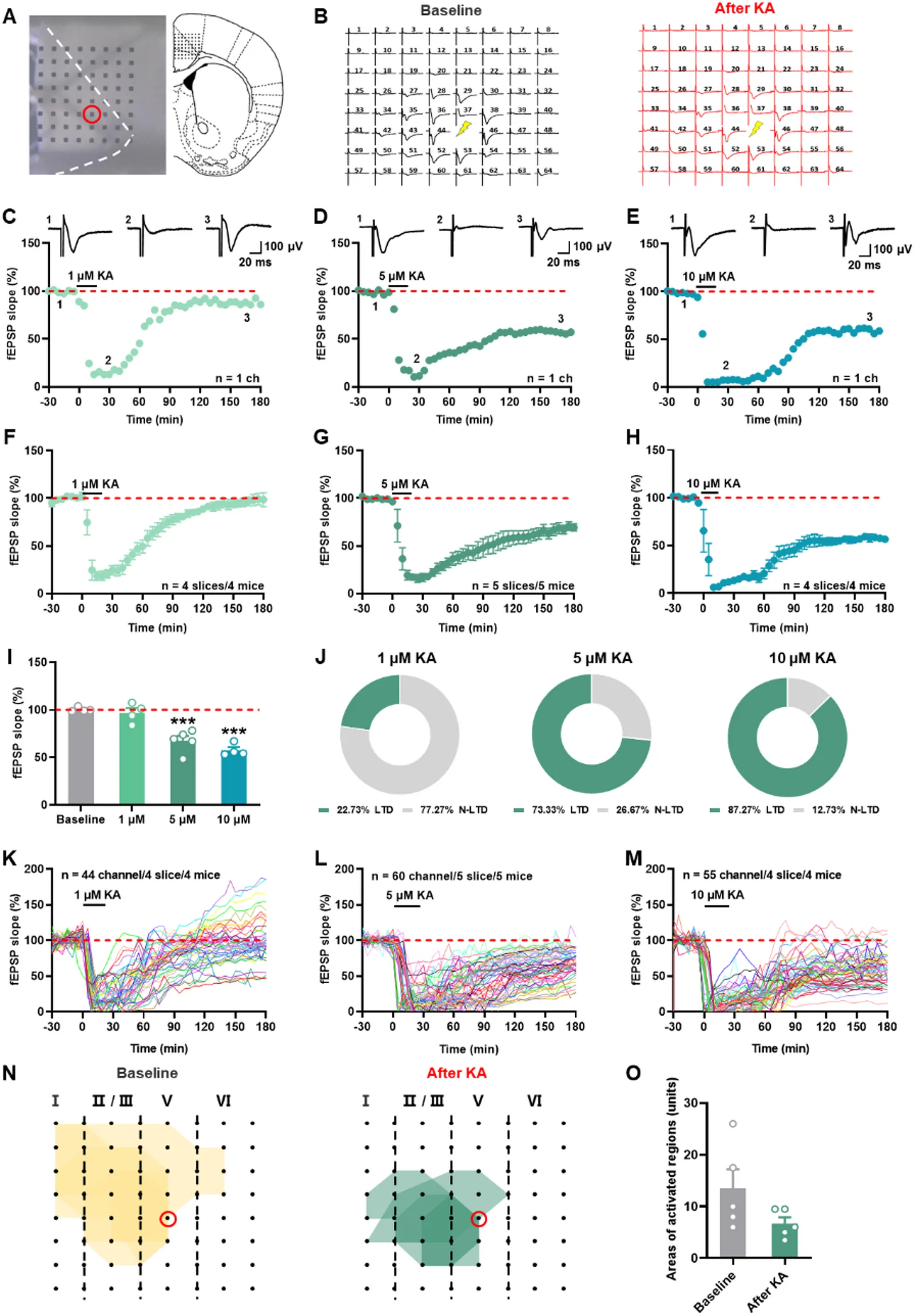
Kainate receptor agonist induced dose-dependent LTD in the ACC. **A**. Schematic of MED64 multielectrode array recording configuration in acute ACC slices. Stimulation was delivered to layer V/VI (red circle) with field excitatory postsynaptic potentials (fEPSPs) recorded from 63 channels. **B**. Representative fEPSP traces demonstrating synaptic depression following 5 µM KA application. Left: baseline; right: post-KA. **C**-**H**. Time-course plots of normalized fEPSP slopes showing: No LTD with 1 µM KA (96.78 ± 5.08% of baseline, *n* = 4 slices/4 mice); Persistent LTD with 5 µM KA (67.47 ± 5.15% of baseline, *n* = 5 slices/5 mice); Robust LTD with 10 µM KA (57.60 ± 3.21% of baseline, *n* = 4 slices/4 mice). **C**, **D**, and **E** were single-channel data. **F**, **G**, and **H** were combined into a single dataset. **I**. Statistical results of KA-induced LTD (final 30 min recording). ***p < 0.001 vs. baseline (one-way ANOVA with Tukey post-hoc, *n* = 4–5 slices/4–5 mice). **J**. Percentage of channels exhibiting LTD across KA concentrations: 1 µM: 22.7% (10/44 channels, *n* = 4 mice); 5 µM: 73.3% (44/60 channels, *n* = 5 mice); 10 µM: 87.3% (48/55 channels, *n* = 4 mice). **K**-**M**. Individual normalized fEPSP slopes from activated channels after KA application. **N**. Spatial distribution of activated (yellow) and LTD-positive (green) channels on the MED64 array. Black dots denote electrode positions; vertical lines demarcate ACC layers. The red circles represent the stimulation sites. **O**. Statistical comparison of areas of activated regions vs. LTD regions, summarizing results from multiple experimental groups

As mentioned earlier [4, 31]. After 1 h of incubation, one slice containing the ACC was placed in a MED64 probe covering most of the 64 electrodes (Fig. 1A). After a slice position was fixed, a mesh and an anchor were carefully placed on the slice to ensure the stability of the slice during the experimental recording process. The MED64 probe was perfused with oxygenated (95% O_2_ and 5% CO_2_) ACSF at 28–30℃ and maintained at a 2–3 mL/min flow rate during the experiment. The slice was allowed to recover for at least 1 h before the start of recording. One planar microelectrode with monopolar constant-current pulses (0.2 ms) was used for stimulation of the ACC slice. The stimulation site was selected within the deep layer V-VI region, and the intensity was adjusted so that a semi-maximum field excitatory postsynaptic potential (fEPSP) was evoked in the channels closest to the stimulation site. The fEPSPs are extracellularly recorded synaptic potentials that reflect the summed activity of a population of synchronously activated excitatory synapses in the local neuronal network. The response was generated and recorded by a data acquisition software (Mobius, Panasonic Alpha-Med Sciences). The channels with fEPSP were considered active channels, and their fEPSP responses were sampled every 1 min and averaged every 5 min when analyzing. The parameter of ‘slope’ indicated the averaged slope of each fEPSP recorded by activated channels. The stable baseline responses were first recorded until the baseline response variation was 5% in most of the active channels within 30 min. After baseline records were stable, the drug KA was added to the perfusion fluid for 20 min before washout. The antagonist or inhibitor was applied 10 min before KA was applied and washed out together with KA. Then the fEPSP responses were recorded for 3 h.

### Whole-cell patch-clamp recording

Whole-cell patch-clamp recordings were based on our previous studies [30, 32]. Experiments were performed in a recording chamber placed in an Olympus BX51W1 microscope with infrared DIC optics for visualization of whole-cell patch-clamp recording. Excitatory postsynaptic currents (EPSCs) and inhibitory postsynaptic currents (IPSCs) were recorded in the voltage-clamp configuration, and the resting membrane potential was held at -60 mV from layer II/III pyramidal neurons in the ACC with an Axon 200B amplifier (Axon Instruments). The recording pipettes (3–5 MΩ) were filled with a solution containing (in mM) 145 K-gluconate, 5 NaCl, 1 MgCl_2_, 0.2 EGTA, 10 HEPES, 2 Mg-ATP, and 0.1 Na_3_-GTP (adjusted to pH 7.2 with KOH, 290 mOsm/L). The patch electrode internal solution containing (in mM) 112 Cs-Gluconate, 5 TEA-Cl, 3.7 NaCl, 0.2 EGTA, 10 HEPES, 2 Mg-ATP, 0.1 Na_3_-GTP, and 5 QX-314 (adjusted to pH 7.2 with CsOH, 290 mOsmol) was used for recording sIPSCs and eIPSCs. KAR-mediated EPSCs were induced by applying repetitive high-frequency stimulation (5 pulses at 200 Hz) in the presence of the selective, non-competitive AMPA receptor antagonist GYKI 53,655 (100 µM). Unlike AMPARs, KARs exhibit slower desensitization kinetics; therefore, during such high-frequency trains, glutamate accumulates in the synaptic cleft and progressively activates postsynaptic KARs. This standard protocol yields a compound KAR-mediated current large enough for reliable measurement [13, 23]. A bipolar tungsten-stimulating electrode was placed in layers V/VI of the ACC to activate afferent fibers projecting to layer II/III pyramidal neurons. The evoked EPSCs were recorded from the postsynaptic neurons in the voltage-clamp configuration. The input (stimulus intensity)-output (EPSC amplitude) (I-O curves) relationships in the ACC pyramidal neurons were recorded at different stimulus intensities. Access resistance was 15–30 MΩ and was monitored throughout the experiment. Data were discarded if access resistance changed by 15% during the experiment. Data were filtered at 1 kHz and digitized at 10 kHz using the Digidata 1440 A. Data were collected and analyzed with Clampex and Clampfit 10.2 software (Axon Instruments).

### Behavioral tests

Von Frey fibers were used to detect the mechanical withdrawal threshold (MWT) of the left hind limb of mice by the Up-Down method as described previously [30, 33]. The mice were placed in a plexiglass cage with a small metal grid at the bottom and were allowed to acclimate for 30 min, and then the von Frey filaments were applied perpendicularly to the plantar surface of the paw until it buckled slightly and was held for 3–6 s. Positive reactions include rapid paw shaking, paw licking, and sudden withdrawal of the hind paw. A series of filaments has different bending forces, including 0.008, 0.02, 0.04, 0.16, 0.4, 0.6, 1, 1.4, and 2.0 g. An initial filament force of 0.4 g was applied to the mice. If a negative response occurred, the next stronger filament was applied. This procedure was continued until at least five responses were collected after the first response change (i.e., the first transition from a positive to a negative response or vice versa). The 50% withdrawal threshold was calculated using the up-down formula and expressed in grams. An interval of at least 5 min was maintained between consecutive tests on the same paw.

According to the previous description [29], the mice were placed on a hot plate at a temperature of 55 ± 1℃. The latency time in the first positive reaction of the hind paws was recorded. Positive responses include rapid paw shaking, paw licking, and jumping. In order to avoid mouse burns, the maximum test time was set to 30 s. The test was repeated three times with an interval of 5 min. The final latency time was the average of the three measurements.

The elevated plus maze (EPM) test was performed as previously described [14, 30]. The EPM consists of two open arms (30 × 6 cm) and two closed arms (30 × 6 cm). The open arms and the closed arms are perpendicular to each other, forming a cross, and the cross part is the central area. For each test, the mouse was placed in the central area and faced one of the open arms. Allowed the mouse to move freely for 5 min. Video cameras were used to record the mouse activity track, and Smart3.0 software (Panlab) was used to analyze the activity of mice.

The open field test was performed as previously described [32]. The device of the open field is a white plastic box of 43 cm × 43 cm × 30 cm. Define the 21.5 × 21.5 cm in the center of the open field as the center zone and the rest as the periphery zone. During the experiment, the mice were placed in the central area and moved freely for 15 min. The video camera was used to record the mouse activity track, and the Smart3.0 software (Panlab) was used to analyze the activity of mice.

### Western blot assay

Mice were deeply anesthetized with isoflurane and rapidly decapitated. The brain was quickly removed from the skull and immediately placed in ice-cold oxygenated ACSF for approximately 1 min to firm the tissue. Coronal brain slices (1 mm thick) containing the ACC were cut using a pre-chilled brain matrix (mouse coronal slice matrix, 1 mm interval) on an ice-cold metal platform. The ACC was then micro-dissected bilaterally from these slices under a dissecting microscope using the following anatomical landmarks: the cingulate cortex region dorsal to the corpus callosum, extending from the midline to approximately 0.5 mm laterally, and spanning from approximately + 2.5 mm to + 1.0 mm anterior to bregma, according to the mouse brain atlas of Paxinos and Franklin. Particular care was taken to exclude the adjacent secondary motor cortex (M2) and prelimbic cortex (PrL) by using the clearly visible midline and corpus callosum as boundaries. The dissected tissue samples were immediately frozen on dry ice and stored at -80 °C until further processing. The tissue samples were homogenized with ice-cold Radioimmunoprecipitation Assay (RIPA) lysis buffer (50 mM Tris, 150 mM NaCl, 1% Nonidet P-40 (NP-40), 0.25% sodium deoxycholate, 1 mM phenylmethylsulphonyl fluoride; pH 7.4) with a protease-/phosphatase-inhibitor cocktail (Sigma Aldrich, Canada) using a tissue homogenizer (Servicebio, Wuhan, China). Membrane proteins were extracted from crude homogenate using a Membrane and Cytoplasmic Protein Extraction Kit (BL671A, Biosharp). Tissues were lysed in lysis buffer with protease inhibitors and dithiothreitol (DTT), then centrifuged to collect the cytoplasmic protein-containing supernatant. The pellet was resuspended in Extraction Buffer, vortexed, and centrifuged to obtain the membrane protein-rich supernatant. Following the measurement of protein concentrations using a Bradford 1× Dye Reagent (Bio-Rad, US), equivalent amounts of protein samples underwent heat denaturation at 70 °C. Subsequently, the denatured samples were separated via 10% sodium dodecyl sulfate-polyacrylamide gel electrophoresis (SDS-PAGE) and transferred to a nitrocellulose membrane. The membrane was blocked with 5% non-fat milk in Tris-buffered saline containing 0.1% Tween-20 (TBST) for 1 h at room temperature. Subsequently, the membranes were immuno-labelled overnight at 4 °C with antibodies of mouse anti-GluK1 (1:1000; Millipore, US), mouse anti-GluK2/3 (1:2000; Millipore, US), and rabbit anti-Na, K-ATPase (1:2000; Beyotime Biotechnology, Shanghai, China). After washing with TBST, the blots were incubated 1 h at room temperature with the horseradish peroxidase-conjugated anti-mouse and anti-rabbit secondary antibodies (1:5000; Millipore, US). The blots were detected with western peroxide reagent and luminol/enhancer reagent (Immobilon Western Chemiluminescent HRP Substrate; EMD Millipore) and captured by the Champchemi System with SageCapture software (Sagecreation Service for Life Science, Beijing, China). The band intensity was quantified and analyzed by ImageJ software. For each sample, the optical density (OD) of the target protein band was first normalized to the OD of the Na, K-ATPase band from the same lane to correct for loading variations: Normalized intensity = OD_target_ / OD_Na, K−ATPase_. For cross-group comparison, these normalized values were then divided by the mean normalized value of the sham-operated group, setting the sham group means to 1: Relative expression = Normalized intensity / Mean (Normalized intensities of sham group). Final results are expressed as fold changes relative to sham controls. Data from three independent biological replicates per group were used for quantification [13].

### Quantitative real-time reverse transcription (RT)–polymerase chain reaction (PCR)

Mice were anesthetized with isoflurane, and the brains were quickly removed. The ACC was micro-dissected and kept in RNAlater (Beyotime Biotechnology, Shanghai, China) for analysis via quantitative real-time reverse transcription (RT)-polymerase chain reaction (PCR). Specifically, tissues were first homogenized in a cold tissue homogenization machine (Servicebio, Wuhan, China), in lysis buffer from the RNAeasy™ RNA extraction kit (Beyotime Biotechnology). Total RNA was then extracted based on the manufacturer’s instructions and quantified using a NanoDrop spectrophotometer (ThermoFisher Scientific, Grand Island, NY). Total RNA at 1000 ng was reverse transcribed using the RevertAid First Strand cDNA Synthesis Kit (ThermoFisher Scientific) according to the manufacturer’s instructions, with oligo (dT) primers. Real-time PCR was performed with a 20 µL reaction with 20 ng of cDNA, 250 nM of forward and reverse primers, and 10 µL of SYBR Green Pro Taq HS Premix (Accurate Biology, Hunan, China) in a BIO-RAD CFX96 real-time PCR system (Bio-Rad Laboratories, Hercules, CA). The primers are listed in Table 1. The cycle parameters consisted of an initial 3 min incubation at 95 ℃, followed by 40 cycles of 95 ℃ for 10 s, 60 ℃ for 30 s, and 72 ℃ for 30 s. The mRNA levels from the control group were averaged, and ratios of mRNA levels in each group to the averaged mRNA levels were calculated using the ΔCt method (2^−ΔΔCt^). All data were normalized to glyceraldehyde-3-phosphate dehydrogenase (*Gapdh*), which was used as an internal control [34].

**Table 1:** Primer sequences for real-time qPCR on *Grik1–5 *

	Forward primers(5‘-3‘)	Reverse primers(5‘-3‘)
*Grik1*	CGCGGCACAGTCCTTATCC	CACTTGAGGGGAGGTCTGA
*Grik2*	ATGGGTTCTCCATATCGAGACA	GGTGGCACTAACCAAATAGAAGA
*Grik3*	AGGTCCTAATGTCACTGACTCTC	TGCCATAAAGGGTCCTATCAGAC
*Grik4*	CCCTGAGGATTGCTGCTATCT	CACCCTTGGGGAGGATCTGA
*Grik5*	ATAGTCGCCTTCGCCAATCC	GTGTCCGTGGTCTCGTACTG
*Gapdh*	TCGGTGTGAACGGATTTGGC	TCCCATTCTCGGCCTTGACT

### Immunofluorescence staining

Following anesthesia, mice were transcardially perfused with 4% paraformaldehyde (PFA). The whole brains were then removed and post-fixed in 4% PFA for 4–6 h at 4 °C. Subsequently, the brains were sequentially dehydrated in 10%, 20%, and 30% sucrose solutions. The dehydrated brains were frozen and sectioned into 20‑µm-thick slices using a cryostat (CM1950, Leica, Germany). The obtained brain sections were washed with 1× PBS and blocked with 10% normal goat serum for 1 h at room temperature. They were then incubated overnight at 4 °C with primary antibodies: rabbit polyclonal anti‑CaMKIIα (1:400; YT0623, Immunoway) and rabbit polyclonal anti‑GAD67 (1:400; 33379, Signalway Antibody). After washing, the sections were incubated with Alexa Fluor 488‑conjugated goat anti‑rabbit IgG (1:1000; A11008, Invitrogen, Thermo Fisher Scientific) for 2 h at room temperature in the dark. Following additional washes and air‑drying, the sections were mounted using an antifade mounting medium containing DAPI (SB0027, Biofount). Images were acquired using a confocal laser scanning microscope (FV3000, Olympus, Japan).

### Statistical analysis

Mice were randomly assigned to experimental groups using a random number generator. Behavioral tests and electrophysiological recordings were performed and analyzed by investigators blinded to the treatment conditions. Sample sizes were determined a priori based on power analysis (80% power, α = 0.05) and our previous experience. No animals or data points were excluded except for predefined criteria (e.g., recording instability or failure to meet baseline stability). The LTD data were collected and analyzed with the MED64 Mobius software, Clampex 10.2, and Clampfit 10.2 software (Molecular Devices). All data were presented as the means ± standard error of the mean (SEM). Statistical comparisons were made with the two-tailed unpaired or paired Student’s *t*-test for comparison between the two groups. For comparison among the three groups, we used one-way ANOVA or two-way ANOVA (Student-Newman-Keuls or Tukey test was used for post-hoc comparisons). All statistical analyses were carried out with GraphPad Prism 8.0 (GraphPad Software). In all cases, *p <* 0.05 was considered statistically significant. The graphical abstract image was created with www.biorender.com.

## Results

### Induction of KAR-mediated LTD in the ACC

Previous studies have established that kainate receptors (KARs) mediate LTD in the hippocampus and perirhinal cortex [17, 19]. Given the known role of KARs in presynaptic LTP within the ACC, we hypothesized that they also mediate LTD in this region [21]. To test this, we employed a 64-channel multielectrode array (MED64) system combined with pharmacological approaches to examine plasticity induction by the KAR agonist kainic acid (KA) in acute ACC slices. As shown in Fig. 1A, stimulation was applied to deep layers V/VI (red circle), and field excitatory postsynaptic potentials (fEPSPs) were recorded from the surrounding 63 channels. The fEPSPs are extracellularly recorded synaptic potentials that reflect the summed activity of a population of synchronously activated excitatory synapses in the local neuronal network. KA (1, 5, and 10 µM) was bath-applied for 20 min after a 30-min stable baseline, resulting in widespread synaptic depression, as indicated by reduced fEPSP amplitudes (Fig. 1B). Dose–response analysis showed that 1 µM KA failed to induce significant LTD, but 5 µM and 10 µM KA induced robust and persistent chemical LTD lasting for more than 3 h (Fig. 1C–H). Statistical quantification revealed a concentration-dependent effect (baseline: 100.4 ± 1.21%; 1 µM: 96.78 ± 5.08%; 5 µM: 67.47 ± 5.15%; 10 µM: 57.60 ± 3.21%; Fig. 1I).

We further analyzed the spatial recruitment of LTD by calculating the ratio of channels exhibiting LTD at varying concentrations of KA. At 1 µM, LTD was observed in only 22.73% (10 out of 44) of activated channels. This proportion significantly increased to 73.33% (44 out of 60) at 5 µM and 87.27% (48 out of 55) at 10 µM (Fig. 1J–M). These results indicate that KA enhances both the amplitude of LTD and the spatial extent of LTD recruitment in a dose-dependent manner within the ACC. To minimize potential excitotoxicity associated with higher concentrations, we selected 5 µM KA for subsequent experiments.

Additionally, MED64 recordings captured “silencing responses” defined as channels that were active during baseline but became quiescent following LTD induction (silenced channels). Similar to low-frequency stimulation (LFS) induced LTD, KA-induced LTD exhibited silencing responses, primarily located at the periphery of the original active area (Fig. 1N, O), with an average of 5–7 silenced channels per slice. Collectively, these data demonstrate that KAR activation effectively induces LTD in the ACC.

### The GluK1 subunit and Ca^2+^-activated PKA signaling pathway are required for KAR-mediated LTD

**Fig. 2:**
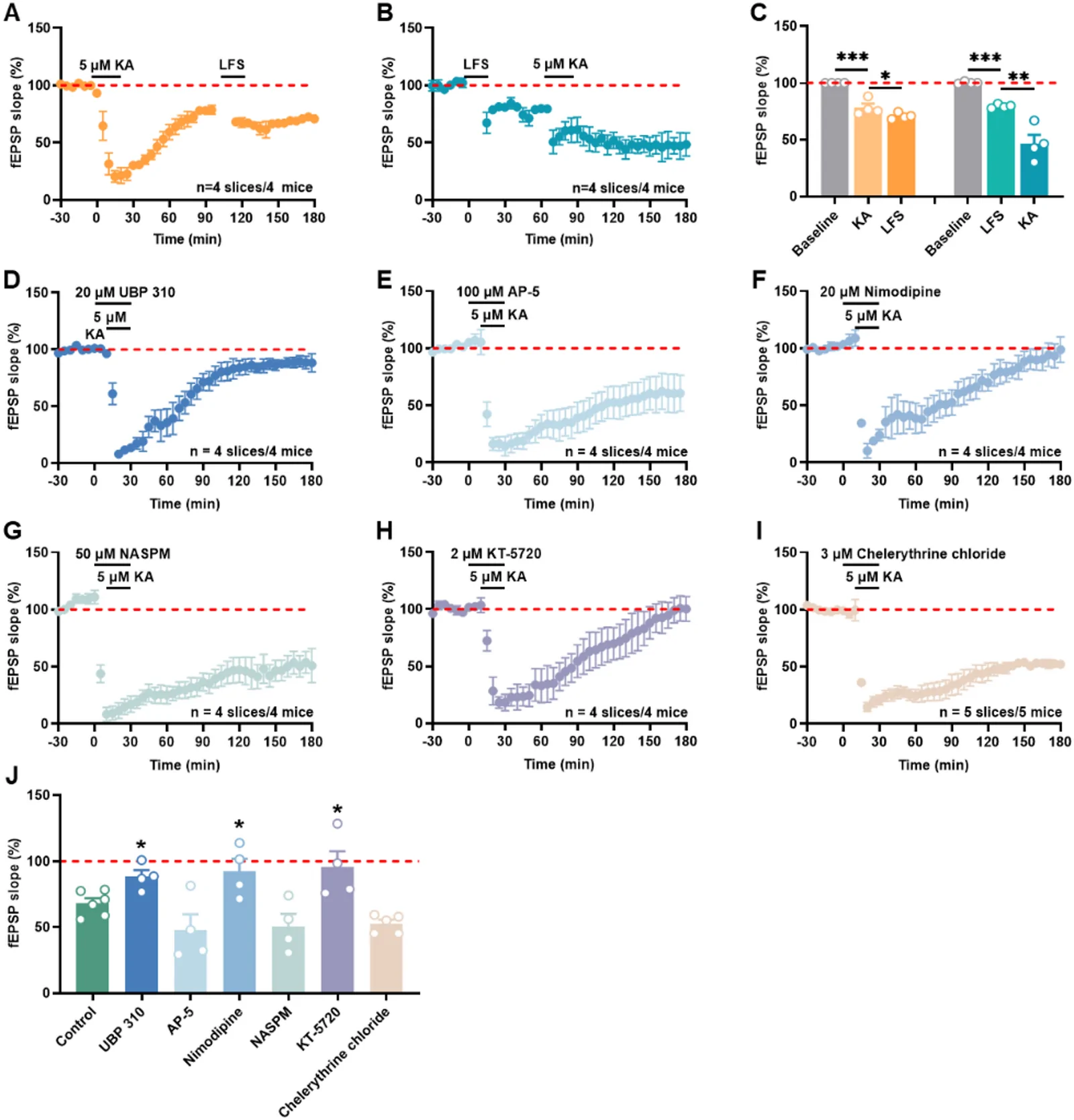
Pharmacological dependence of KAR-mediated LTD in the ACC. **A**. Time-course plots of normalized fEPSP slopes showing: post-KA LTD stabilization (100 min), low-frequency stimulation (LFS, 1 Hz/15 min) successfully induced additional LTD (70.89 ± 1.35 vs. 78.3 ± 3.37 of KA-LTD, *n* = 4 slices /4 mice). **B**. Time-course plots of normalized fEPSP slopes showing: prior LFS-induced LTD did not preclude subsequent KAR-mediated LTD (46.61 ± 7.61 vs. 79.59 ± 0.85 of LFS-LTD, *n* = 4 slices /4 mice). **C**. Statistical comparison of KA-induced LTD and LFS-induced LTD. *n* = 4 slices/4 mice, *p < 0.05, **p < 0.01, ***p < 0.001 vs. baseline (one-way ANOVA with Tukey post-hoc). **D**-**I**. Time-course plots showing blockade of KA-induced LTD by GluK1 antagonist UBP310 (20 µM) (88.20 ± 4.96%, *n* = 4 slices/4 mice), L-VGCC blocker Nimodipine (20 µM) (92.25 ± 9.50%, *n* = 4 slices/4 mice), PKA inhibitor KT-5720 (2 µM) (95.40 ± 12.11%, *n* = 4 slices/4 mice). KA-induced LTD can not be blocked by NMDA receptor antagonist AP-5 (100 µM) (47.74 ± 11.95%, *n* = 4 slices/4 mice), CP-AMPAR antagonist NASPM (50 µM) (50.48 ± 9.44%, *n* = 4 slices/4 mice), or PKC inhibitor chelerythrine chloride (3 µM) (52.55 ± 3.07%, *n* = 5 slices/5 mice). **J**. Summary data of normalized fEPSP slopes (final 30 min). *p < 0.05 (one-way ANOVA with Tukey post-hoc, *n* = 4–6 slices/4–6 mice)

To determine whether KA-induced LTD and low-frequency stimulation (LFS)-induced LTD operate via distinct mechanisms, we sequentially applied both induction protocols in the ACC. Following the stabilization of KA-induced LTD (100 min), subsequent LFS (1 Hz, 15 min) successfully elicited additional LTD, sustaining synaptic depression for over 90 min (Fig. 2A, C; *p < 0.05). Conversely, KA application induced robust LTD even after the establishment of LFS-induced LTD (Fig. 2B, C; **p < 0.01). The absence of mutual occlusion suggests that KA- and LFS-induced LTD may coexist via independent mechanisms within the ACC.

Subsequently, we are attempting to uncover the molecular mechanisms underlying KA-induced LTD. Following a 30-min stable baseline recording, specific antagonists or inhibitors were applied 10 min before KA (5 µM) perfusion (20 min) and washout, with responses monitored continuously for 2.5 h (Fig. 2D–I). Initially, to assess the dependence on kainate receptors (KARs), we applied the GluK1 antagonist UBP310 (20 µM). This treatment significantly abolished KA-induced LTD (88.20 ± 4.96% of baseline; Fig. 2D), confirming that the LTD is KAR-dependent. We then examined potential overlaps with canonical NMDAR- and Ca^2+^-dependent LTD pathways. The NMDA receptor antagonist AP-5 (100 µM) failed to prevent the fEPSP depression induced by KA (47.74 ± 11.95% of baseline; Fig. 2E). Instead, Nimodipine (20 µM), an L-type voltage-gated calcium channel (L-VGCC) blocker, prevented the induction of KA-induced LTD (92.25 ± 9.50% of baseline; Fig. 2F). In addition, the calcium-permeable AMPA receptor (CP-AMPAR) antagonist Naspm (50 µM) had no effect on KA-induced LTD (50.48 ± 9.44% of baseline; Fig. 2G). These findings together demonstrate that KA-induced LTD in the ACC requires activation of GluK1 receptors and L-VGCCs but not NMDARs and CP-AMPARs.

Given the established roles of Ca^2+^-activated protein kinase A (PKA) and protein kinase C (PKC) in ACC synaptic plasticity, we probed their specific contributions. The PKA inhibitor KT-5720 (2 µM) significantly attenuated KA-induced LTD (95.40 ± 12.11% of baseline; Fig. 2H), whereas the PKC inhibitor chelerythrine chloride (3 µM) had no effect (52.55 ± 3.07% of baseline; Fig. 2I). Taken together, these data demonstrate that the Ca^2+^-activated PKA signaling pathway is required for KA-mediated LTD in the ACC.

### The GluK1-selective agonist ATPA induces KAR-mediated LTD in the ACC

**Fig. 3:**
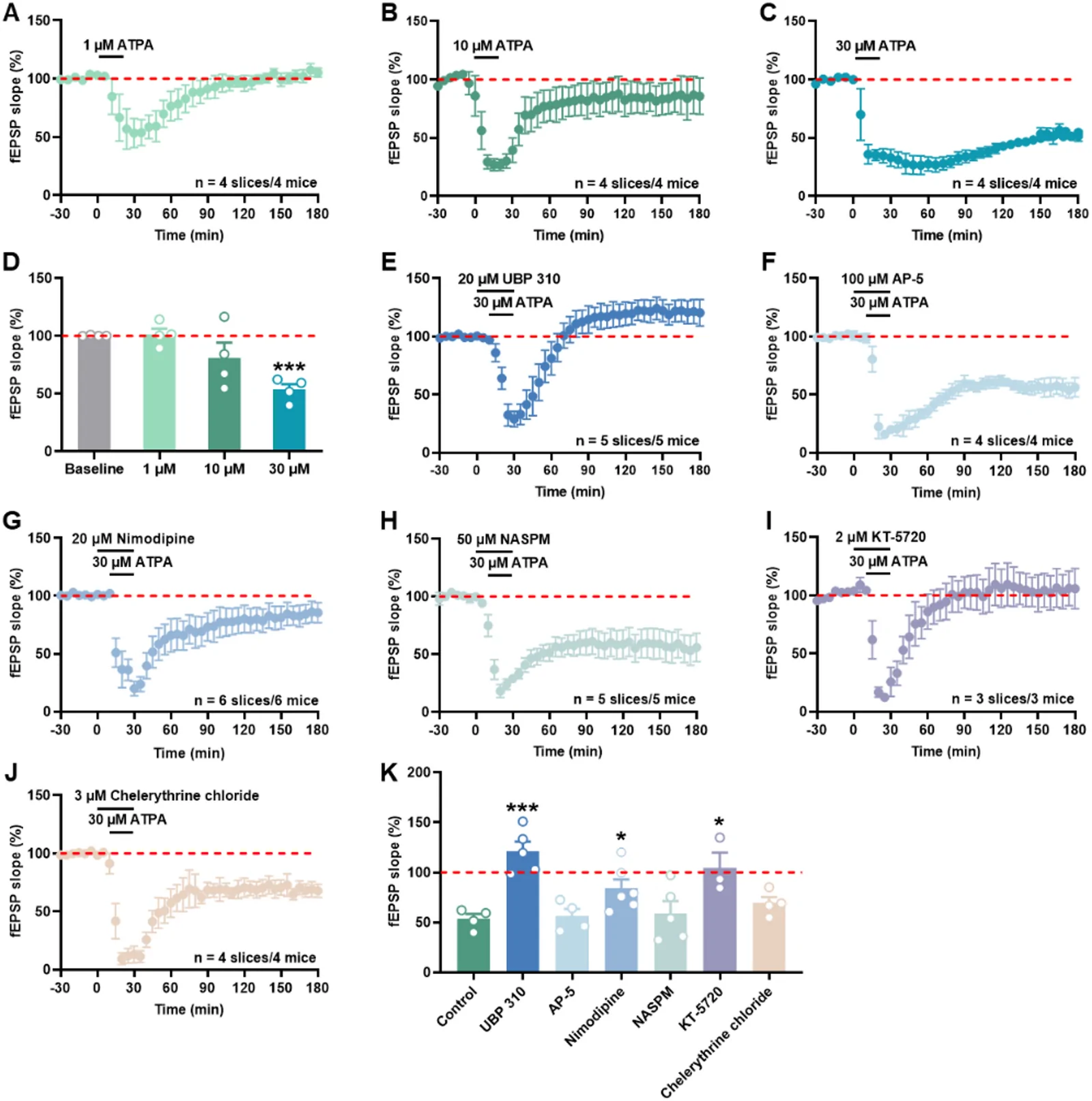
GluK1-selective agonist ATPA induced LTD in the ACC. **A**-**C**. Time-course plots showing: No LTD with 1 µM ATPA (101.4 ± 5.1%, *n* = 4 slices/4 mice), Marginal LTD with 10 µM ATPA (80.8 ± 13.4%, *n* = 4 slices/4 mice), Significant LTD with 30 µM ATPA (53.4 ± 5.0%, *n* = 4 slices/4 mice). **D**. Summary of normalized fEPSP slopes (final 30 min). ***p < 0.001 vs. baseline (one-way ANOVA with Tukey post-hoc, *n* = 4 slices/4 mice). **E**-**J**. ATPA-induced LTD (30 µM) was blocked by antagonists targeting GluK1(121.10 ± 9.70%, *n* = 5 slices/5 mice), L-VGCC (83.94 ± 9.04%, *n* = 6 slices/6 mice), and PKA (104.30 ± 15.50%, *n* = 3 slices/3 mice). But was unaffected by NMDA (56.19 ± 7.34%, *n* = 4 slices/4 mice), CP-AMPA (59.14 ± 12.24%, *n* = 5 slices/5 mice), PKC blockers (69.12 ± 6.25%, *n* = 4 slices/4 mice). **K**. Summary data of normalized fEPSP slopes (final 30 min). *p < 0.05, ***p < 0.001 vs. ATPA alone (one-way ANOVA with Tukey post-hoc, *n* = 3–6 slices/ 3–6 mice)

To further validate the specific involvement of the GluK1 subtype in KAR-mediated LTD, we employed the GluK1-selective agonist ATPA. Consistent with the profile of KA-induced LTD, bath perfusion of ATPA (20 min) elicited LTD in ACC brain slices in a dose-dependent manner (1 µM: 101.4 ± 5.1% of baseline; 10 µM: 80.8 ± 13.4% of baseline; 30 µM: 53.4 ± 5.0% of baseline; Fig. 3A-D, ***p < 0.001). These results demonstrate that the direct activation of GluK1 receptors is sufficient to induce KAR-dependent LTD.

Subsequently, we examined whether the molecular mechanisms underlying ATPA-induced LTD parallel those of KA-induced LTD. As shown in Fig. 3E, the GluK1 antagonist UBP310 effectively abolished ATPA-induced LTD (121.10 ± 9.70% of baseline; ***p < 0.001). Similarly, the L-VGCC blocker Nimodipine and the PKA inhibitor KT-5720 prevented the induction of LTD following ATPA application (Nimodipine: 83.94 ± 9.04% of baseline; KT-5720: 104.30 ± 15.50%; Fig. 3G and I; *p < 0.05). In contrast, ATPA-induced LTD was not affected by the NMDA receptor antagonist AP-5, the CP-AMPAR antagonist Naspm, and the PKC inhibitor chelerythrine chloride (AP-5: 56.19 ± 7.34% of baseline; Naspm: 59.14 ± 12.24%; chelerythrine chloride: 69.12 ± 6.25%; Fig. 3F, H and J). Thus, these data together show that ATPA induces KAR-mediated LTD that is dependent on the GluK1 subunit and the Ca^2+^-activated PKA signaling pathway.

#### KAR-mediated LTD recorded in whole-cell patch configuration

**Fig. 4:**
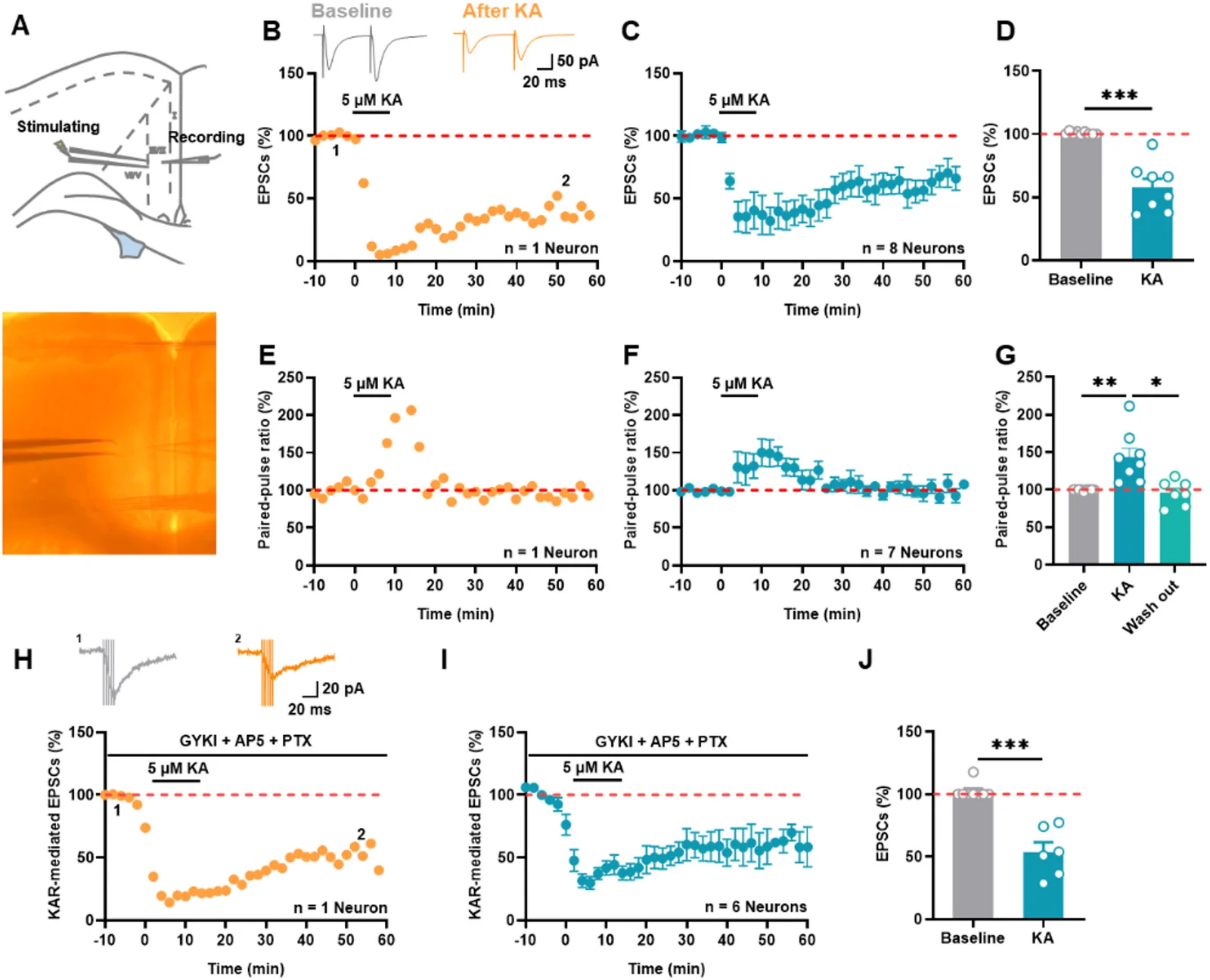
KAR-mediated LTD recorded by the whole-cell patch system in the ACC neuron. **A**. Schematic diagram and photograph showing the placement of the stimulating electrode in layer V/VI of the ACC and the recording electrode in layer V/VI of the ACC. **B**, **C**. Representative traces with an interval of 50 ms recorded in the ACC. Time-course plots of normalized KAR-mediated EPSCs (B: single cell; C: pooled data, *n* = 8 neurons/6 mice). **D**. Summary of normalized KAR-mediated EPSCs (57.94 ± 6.74 vs. 100.8 ± 0.40 of baseline, *n* = 8 neurons/6 mice), ***p < 0.001 vs. baseline by two-tailed unpaired Student’s t-test. **E**-**G**. Paired-pulse ratio (the ratio of EPSC2/EPSC1) was recorded at intervals of 50 ms (*n* = 7 neurons/6 mice; *p < 0.05, **p < 0.01 vs. baseline, one-way ANOVA with Tukey post-hoc). **H**-**J**. KAR-mediated EPSCs were recorded in the presence of GYKI (100 µM), AP-5 (50 µM), and PTX (100 µM). After the perfusion of KA (5 µM) for 10 min, the KAR-mediated LTD could be observed. (**H**: Representative traces and time course points of KAR-mediated LTD of a single cell. **I**: Summarize time course points of KAR-mediated LTD. **J**: statistical results showed the KAR-mediated EPSCs (54.33 ± 8.27 vs. 102.2 ± 2.23 of baseline, *n* = 6 neurons/4 mice, ***p < 0.001, vs. baseline by two-tailed unpaired Student’s t-test)

To complement the findings from field potentials, we used whole-cell patch-clamp recordings to investigate KAR-mediated LTD in individual layer II/III pyramidal neurons of the ACC. A bipolar tungsten-stimulating electrode was positioned in layers V/VI of the ACC to activate afferent fibers that project to layer II/III pyramidal neurons. We recorded the evoked excitatory postsynaptic currents (EPSCs) from the postsynaptic neurons in a voltage-clamp configuration (Fig. 4A). After establishing a stable baseline for 10 min, the application of KA at a concentration of 5 µM for 10 min resulted in a significant and sustained reduction of EPSCs that lasted for over one hour (57.94 ± 6.74% of baseline; Fig. 4B–D), confirming the occurrence of KA-induced chemical LTD at the single-neuron level. To explore potential presynaptic contributions, we assessed the paired-pulse ratio (PPR), which is the ratio of the amplitude of the second EPSC to that of the first EPSC following two identical stimuli delivered at a short interval (e.g., 50 ms). A change in PPR indicates a presynaptic alteration in neurotransmitter release probability. We observed an increase in PPR during KA perfusion, which subsequently returned to baseline levels after washout (Fig. 4E–G). This transient modulation implies that a decrease in presynaptic release plays a role in the induction phase of KAR-mediated LTD.

Given that LTD of KAR-mediated EPSCs (EPSC_KA_ LTD) has been previously demonstrated in the perirhinal cortex, we sought to determine if this form of plasticity is also present in the ACC. EPSC_KA_ were pharmacologically isolated in the presence of picrotoxin (100 µM), AP-5 (50 µM), and the potent AMPA receptor antagonist GYKI 53,655 (100 µM) and were evoked using high-frequency stimulation (five stimuli at 200 Hz). The KA perfusion (5 µM, 10 min) effectively induced robust LTD of EPSC_KA_ in ACC neurons (54.33 ± 8.27% of baseline, ***p < 0.001, Fig. 4H-J). Together, these results indicate that both KAR-mediated LTD of AMPA receptor (AMPAR) currents and LTD of KAR currents coexist in the ACC.

### Involvement of GABA_A_ receptor-mediated inhibitory synaptic transmission in KAR-mediated LTD

**Fig. 5:**
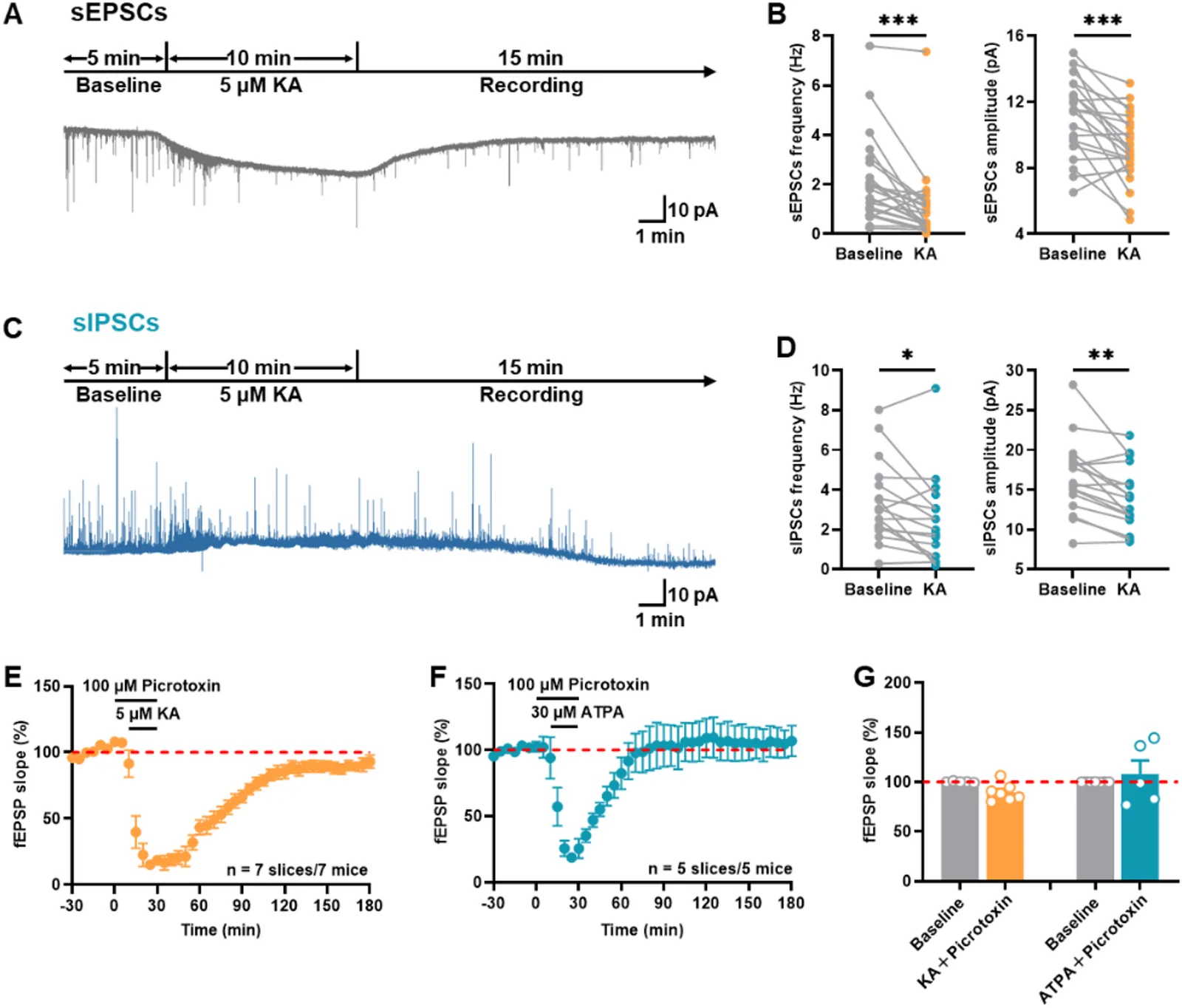
The GABA_A_ receptor was involved in KAR-mediated LTD. **A**, **C**. Representative traces of the sEPSCs and sIPSCs recorded in the ACC neurons. **B**, **D**. Statistical results of frequency (left) and amplitude (right) of the sEPSCs (*n* = 23 neurons/10 mice) and the sIPSCs (*n* = 18 neurons/8 mice). *p < 0.05, **p < 0.01, ***p < 0.001 vs. baseline by two-tailed paired Student’s t-test. **E**, **F**. Summarized time-course plots showing blockade of KA-induced LTD (5 µM) (*n* = 7 slices/7 mice) and ATPA-induced LTD (30 µM) (*n* = 5 slices/5 mice) by GABA_A_ receptor antagonist picrotoxin (100 µM). **G**. Statistical results showed that the KA-induced LTD (5 µM) and ATPA-induced LTD (30 µM) were blocked by the GABA_A_ receptor antagonist picrotoxin. *p* > 0.05 vs. baseline

Earlier studies have demonstrated that KARs have a bidirectional modulation of excitatory and inhibitory synaptic transmission in the ACC [15]. We first tested the effects of KA on spontaneous excitatory (sEPSC) and inhibitory postsynaptic currents (sIPSC) to study a role for inhibitory transmission in the induction of KAR-mediated LTD. Bath perfusion with KA significantly attenuated both the frequency and amplitude of sEPSCs (1.07 ± 0.31 Hz vs. 2.13 ± 0.36 Hz at baseline; 9.22 ± 0.45 pA vs. 11.21 ± 0.50 pA at baseline, ***p < 0.001; Fig. 5A and B), although short-term perfusion does increase the release frequency. Similarly, KA application diminished the frequency and amplitude of sIPSCs (2.45 ± 0.53 Hz vs. 3.37 ± 0.50 Hz at baseline; 14.05 ± 0.97 pA vs. 16.59 ± 1.12 pA at baseline; **p < 0.01; Fig. 5C and D), although it also showed a short-term increase in the release frequency. These results indicate that KA modulation involves a concurrent reduction in both excitatory and inhibitory synaptic drive in the ACC.

Subsequently, we investigated the specific contribution of GABA receptors to KAR-mediated LTD. In field potential recordings, the administration of the non-competitive GABA_A_ receptor antagonist picrotoxin (100 µM) effectively abolished both KA-induced LTD (89.65 ± 3.36% of baseline) and ATPA-induced LTD (107.9 ± 13.82% of baseline; Fig. 5E–G). These findings suggest that functional GABA_A_ receptor activity participates in the induction of KAR-mediated LTD.

#### KAR-mediated LTD is impaired in neuropathic pain mice

**Fig. 6:**
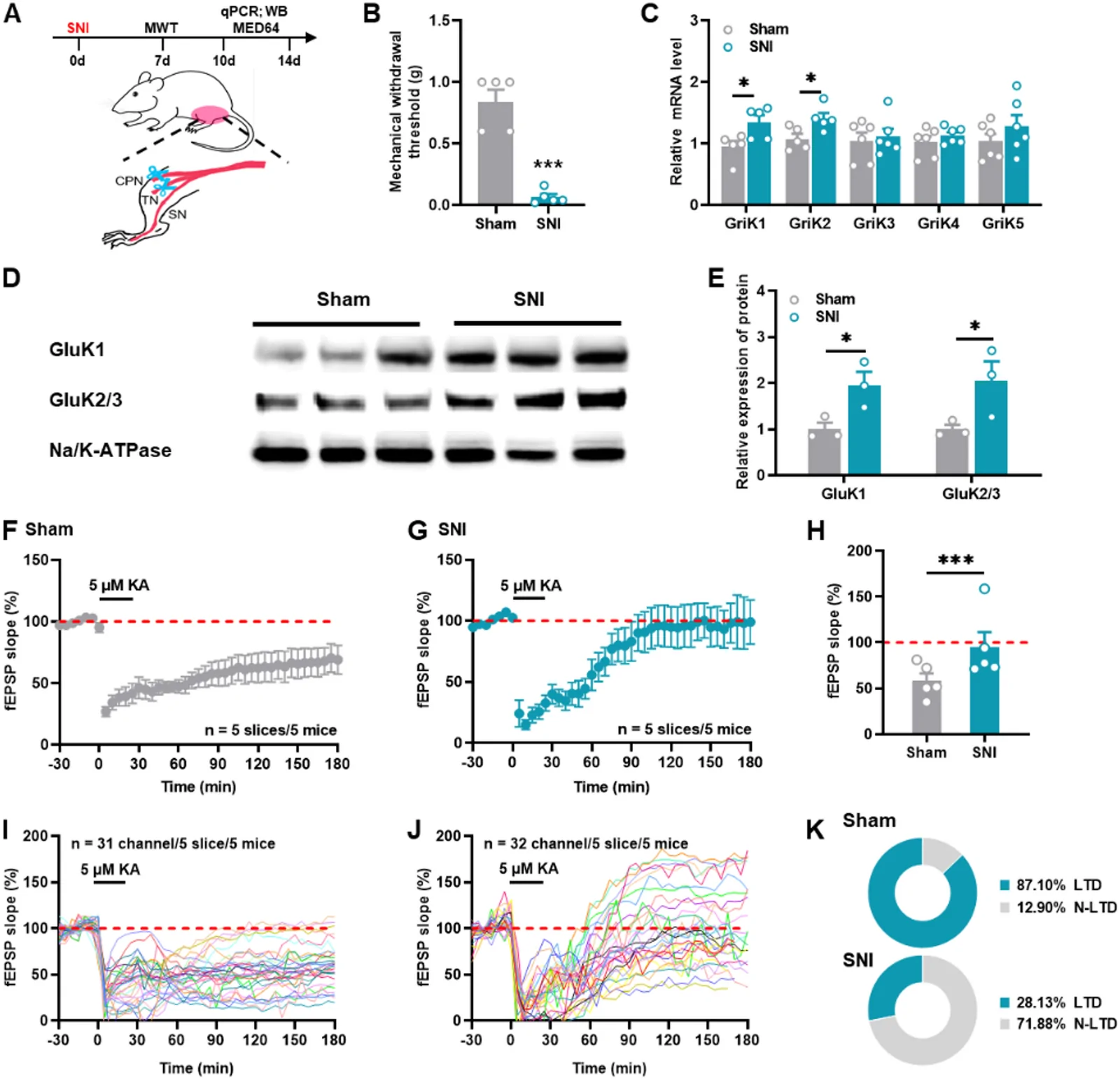
KAR-mediated LTD was impaired in the SNI model mice. **A**. Schematic diagram of SNI surgery: ligation and transection of the common peroneal nerve (CPN) and tibial nerve (TN), preservation of the sural nerve (SN). **B**. Mice in the SNI group had a significantly lower mechanical withdrawal threshold after 7 days of the pain model (0.066 ± 0.025 g vs. 0.84 ± 0.098 g in the sham group, *n* = 5 mice, ***p < 0.001 vs. sham by two-tailed unpaired Student’s t-test). **C**. The mRNA levels of *Grik1* and *Grik2/3* were upregulated in the ACC of SNI mice compared to those of Sham mice (*n* = 5–6 mice, *p < 0.05 vs. Sham by two-tailed unpaired Student’s t-test). **D**. Representative western blot of GluK1 and GluK2/3 expression in the ACC of sham and SNI mice. **E**. GluK1 and GluK2/3 expression were significantly greater in the ACC of SNI mice than in that of sham mice (*n* = 3 mice, *p < 0.05 vs. sham by two-tailed unpaired Student’s t-test). **F**, **G**. The administration of KA-induced LTD was occluded in the ACC slices in SNI mice, but normal in sham mice (*n* = 5 slices/5 mice). **H**. Statistical data of fEPSP slope in sham and SNI mice (*n* = 5 slices/5 mice, ***p < 0.001 vs. sham by two-tailed unpaired Student’s t-test). **I**-**K**. Time-course plots of fEPSP slope in single channel and percentage of channels exhibiting LTD: Sham: 87.10% (27/31 channels, *n* = 5 slices/5 mice), SNI: 28.13% (9/32 channels, *n* = 5 slices/5 mice)

Increasing evidence suggests that both LTD and KAR are involved in regulating nociception [4, 5, 27]. Consequently, we investigated whether KAR-mediated LTD is involved in the pathophysiology of chronic pain. The spared nerve injury (SNI) model was established to induce neuropathic pain in mice (Fig. 6A). The mechanical withdrawal thresholds were significantly reduced in SNI mice after the 7th day compared with sham controls (0.066 ± 0.025 g vs. 0.84 ± 0.098 g, ***p < 0.001), indicating successful induction of neuropathic pain, as shown in Fig. 6B. First, we investigated the possible changes in the expression of KAR subunits in the ACC after nerve injury. RT-qPCR and Western blot analyses were performed in ACC tissues of SNI and sham mice. The mRNA expressions of *Grik1* (encoding GluK1) and *Grik2* (encoding GluK2) were significantly up-regulated in SNI mice compared with those in sham mice. In contrast, *Grik3*, *Grik4*, and *Grik5* mRNA levels were unchanged (Fig. 6C). Western blot analysis corroborated these findings, revealing markedly elevated GluK1 and GluK2/3 protein expression in the ACC of SNI mice (Fig. 6D, E). Collectively, these data suggest that peripheral nerve injury induces selective up-regulation of GluK1 and GluK2/3 subunits in the ACC and suggest a potential aberrant activation of KAR signalling pathways following neuropathic injury.

Subsequently, we investigated KAR-mediated LTD in SNI mice utilizing the MED64 multi-channel recording system. In sham mice, KA (5 µM) elicited persistent LTD (58.19 ± 8.23% of baseline; Fig. 6F, H), with 87.10% of activated channels demonstrating LTD lasting more than three hours (27 of 31 channels; Fig. 6I, K). Conversely, LTD induction was markedly occluded in SNI mice (93.49 ± 15.08% of baseline; Fig. 6G, H), with merely 28.13% of activated channels exhibiting LTD (9 of 32 channels; Fig. 6J, K). The results demonstrate that KAR-mediated LTD is functionally compromised in the ACC of neuropathic pain mice.

### Enhanced KAR-mediated transmission and altered E/I balance in SNI mice

**Fig. 7:**
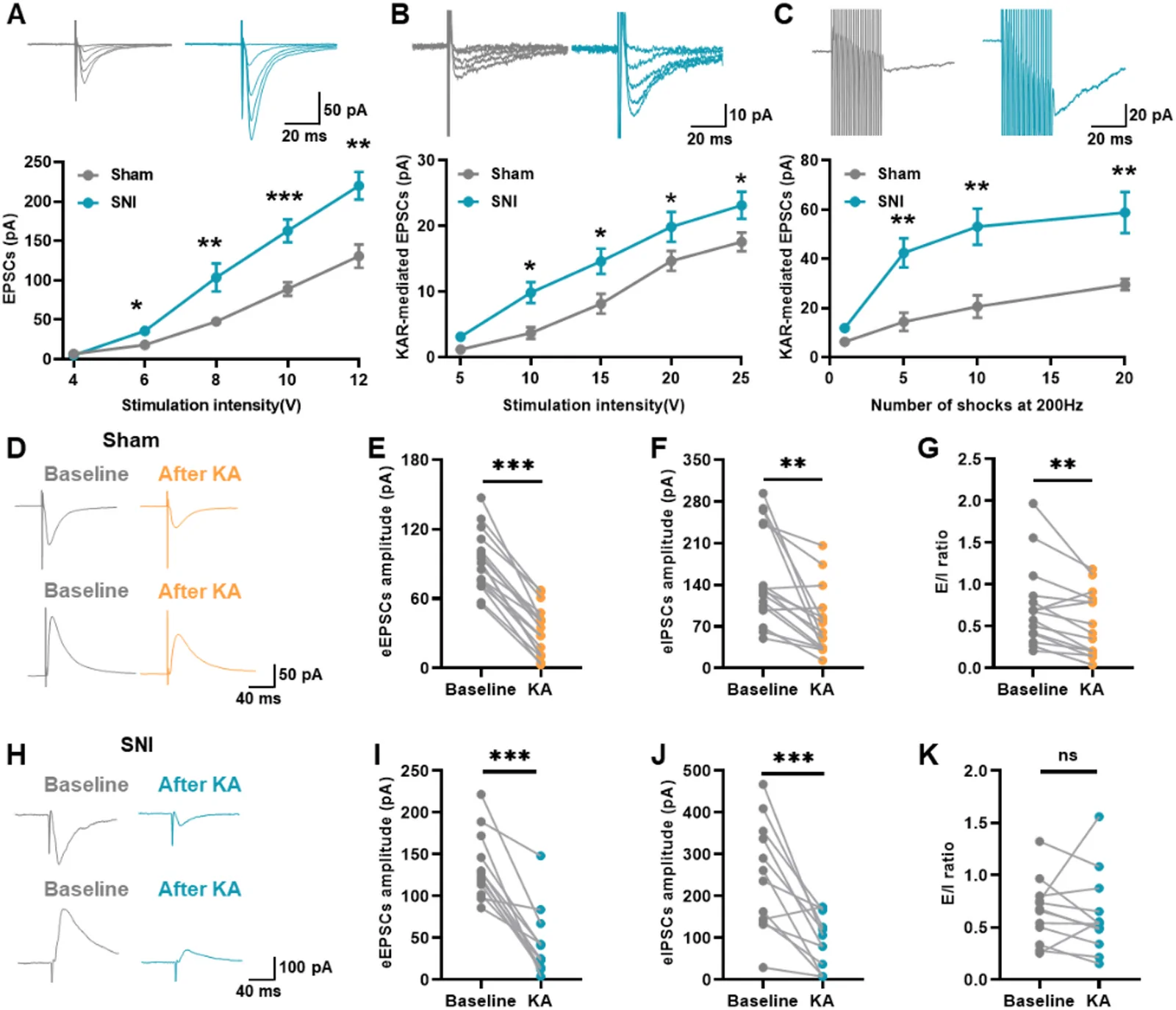
The KAR-mediated EPSCs and the effect of KA on E/I Balance in SNI Mice. **A**. Sample traces and pooled data showed the increased EPSCs in the ACC of SNI mice compared with those in sham mice (SNI: *n* = 8 neurons/4 mice; sham: *n* = 8 neurons/4 mice, *p < 0.05, **p < 0.01, ***p < 0.001 vs. sham, two-way ANOVA with Tukey post-hoc). **B**. Sample traces and pooled data showed the KAR-mediated EPSCs were potentiated in the ACC of SNI mice in the presence of GYKI 53,655 (SNI: *n* = 14 neurons/7 mice; sham: *n* = 8 neurons/4 mice, *p < 0.05 vs. sham, two-way ANOVA with Tukey post-hoc). **C**. Representative traces of KAR-mediated EPSCs were obtained after the application of stimulation (20 shocks at 200 Hz). Statistical results showed that the peak amplitude of the KAR-mediated EPSCs in the ACC of SNI mice was greater than that of Sham mice by repetitive stimulations (1, 5, 10, and 20 shocks) at 200 Hz (SNI: *n* = 13 neurons/5 mice; sham: *n* = 10 neurons/4 mice. *p < 0.05, **p < 0.01 vs. sham, two-way ANOVA with Tukey post-hoc). **D**, **H**. Representative traces of KA (5 µM) reduced evoked excitatory postsynaptic currents (eEPSCs) and evoked inhibitory postsynaptic currents (eIPSCs) in the ACC neurons of sham and SNI mice. **E**, **F**. Statistical results showed that the amplitude of the eEPSCs and the eIPSCs was reduced by application of KA in the sham mice (*n* = 17 neurons/7 mice) **p < 0.01, ***p < 0.001. **G**. Statistical results of the effect of KA on E/I ratio (the ratio of eEPSCs / eIPSCs) in sham mice, **p < 0.01. **I**, **J**. Statistical results showed that the amplitude of the eEPSCs and the eIPSCs was reduced by application of KA in the SNI mice (*n* = 13 neurons/6 mice), **p < 0.01, ***p < 0.001 vs. baseline by two-tailed paired Student’s t-test. **K**. Statistical results of the effect of KA on E/I ratio (the ratio of eEPSCs / eIPSCs) in SNI mice, *p* > 0.05 vs. baseline by two-tailed paired Student’s t-test

We want to clarify the processes responsible for the dysfunction of KAR-mediated LTD in SNI mice. Initially, we delineated the input-output (I-O) connection of basal synaptic transmission in the ACC (Fig. 7A). The I-O curve delineates the correlation between escalating stimulus intensities (input) and the resultant amplitudes of elicited synaptic responses (output), indicating the fundamental strength of synaptic transmission. In accordance with prior research, the I-O curve for EPSCs in SNI mice demonstrated a notable leftward shift relative to sham controls, signifying enhanced basal excitatory synaptic transmission [35]. Subsequently, we broadened this research to include KAR-mediated EPSCs in the SNI mice. Likewise, the amplitude of KAR-mediated EPSCs was increased, and the associated I-O curve was displaced to the left in SNI animals compared to sham mice (Fig. 7B). To measure the total amplitude of KAR-mediated EPSCs, stimulus trains of 1, 5, 10, and 20 were administered at 200 Hz in the presence of GYKI 53,655. Figure 7C illustrates that KAR-mediated EPSC amplitudes were markedly elevated in SNI mice relative to sham mice under repetitive stimulation protocols. The data confirm that KAR-mediated excitatory synaptic transmission is enhanced after nerve injury.

Given that KAR-mediated LTD involves both excitatory and inhibitory components, we investigated whether KA application alters the excitation/inhibition (E/I) balance in SNI mice. Evoked EPSCs (eEPSCs) and IPSCs (eIPSCs) were recorded from the same ACC neurons before and after KA application (Fig. 7D, H). In sham mice, KA perfusion significantly suppressed both eEPSC and eIPSC amplitudes (eEPSCs: 91.51 ± 6.11 pA vs. 32.76 ± 5.08 pA, ***p < 0.001; eIPSCs: 154.0 ± 20.17 pA vs. 75.03 ± 13.88 pA, **p < 0.01; Fig. 7E, F). Importantly, the E/I ratio (eEPSC/eIPSC amplitude) was significantly reduced after KA perfusion (0.73 ± 0.13 vs. 0.52 ± 0.10, *p* < 0.01, Fig. 7G), supporting a preferential inhibitory effect of KA on excitatory synaptic transmission in sham mice. In SNI mice, while KA perfusion similarly attenuated the amplitudes of eEPSCs and eIPSCs (eEPSCs: 135.7 ± 11.61 pA vs. 44.04 ± 11.57 pA, ***p < 0.001; eIPSCs: 247.0 ± 37.65 pA vs. 89.99 ± 18.42 pA, ***p < 0.001; Fig. 7I and J), the E/I ratio remained unchanged (0.65 ± 0.09 vs. 0.62 ± 0.11, *p* > 0.05, Fig. 7K). Collectively, these findings indicate that the KA-induced shift toward inhibition is compromised in the ACC of neuropathic pain mice.

### Cell-type-specific modulation of GluK1 in excitatory and inhibitory neurons alleviates neuropathic pain and anxiety

**Fig. 8:**
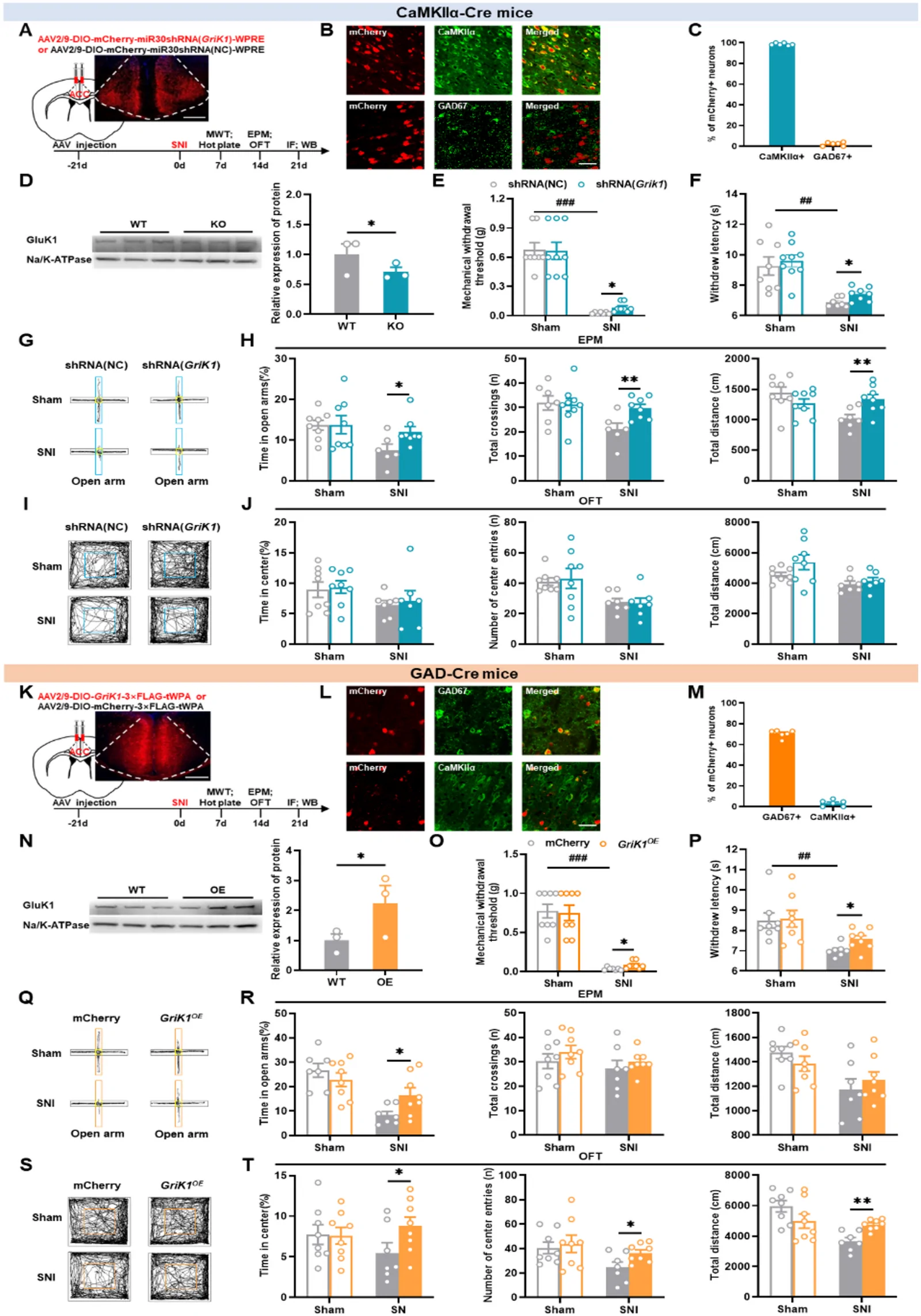
Viral manipulation of the GluK1 receptor attenuated pain and anxiety-like behavior. **A**, **K**. Schematic diagram and timeline of the virus micro-injected into the ACC and the fluorescence verification, scale bar: 500 μm. **B**. Cell-type specificity of viral-mediated GluK1 knockdown in the ACC of CaMKIIα-Cre mice. Representative confocal images showing co-localization of mCherry (red, virus expression) with the excitatory neuron marker CaMKII (green) or the inhibitory neuron marker GAD67 (green) in the ACC. Scale bar: 50 μm. **C**. Quantification showed that 98.57 ± 0.48% of mCherry‑positive neurons co‑expressed the excitatory neuron marker CaMKII, whereas only 2.18 ± 0.73% co‑expressed the inhibitory neuron marker GAD67. (*n* = 6 slices/3 mice). D. Representative Western blot (left) and quantification (right) showing a significant reduction of GluK1 protein expression in the shRNA(*Grik1*) group compared to the shRNA(NC) control group (*n* = 3 mice, *p < 0.05, Na, K-ATPase was used as a loading control). E. Viral injection knockdown of GluK1 in the ACC of CaMKIIα-Cre mice, the mechanical withdrawal threshold of the shRNA(*Grik1*) SNI group in the von Frey test significantly increased (0.09 ± 0.02) compared to the shRNA(NC) SNI group (0.05 ± 0.01, *n* = 8–9 mice, *p < 0.05 vs. shRNA(NC) SNI group, ^###^*p* < 0.001 vs. shRNA(NC) sham group by two-tailed unpaired Student’s t-test). **F**. Viral injection knockdown of GluK1 in the ACC of CaMKIIα-Cre mice, the withdrawal latency in the hot plate test of shRNA(*Grik1*) SNI group significantly prolonged (7.42 ± 1.56) compared to the shRNA(NC) SNI group (6.88 ± 0.12, *n* = 8–9 mice, *p < 0.05). **G**, **Q**. Elevated plus Maze (EPM) trajectory diagram. **H**. The percentage of time in open arms, number of total crossings, and total distance in the EPM were significantly increased in shRNA(*Grik1*) SNI mice, *n* = 7–8 mice, *p < 0.05, **p < 0.01 vs. shRNA(NC) SNI group by two-tailed unpaired Student’s t-test. **I**, **S**. Open Field Test trajectory diagram. **J**. The percentage of time in the central, the number of central entries, and the total distance in the OFT were not significantly different in the shRNA(*Grik1*) SNI mice, *n* = 7–8 mice, *p* > 0.05 vs. the shRNA(NC) SNI group by two-tailed unpaired Student’s t-test. **L**. Cell-type specificity of viral-mediated GluK1 overexpression in the ACC of GAD-Cre mice. Representative confocal images showing co-localization of mCherry (red, virus expression) with the inhibitory neuron marker GAD67 (green) or the excitatory neuron marker CaMKII (green) in the ACC. Scale bar: 50 μm. **M**. Quantification revealed that 70.45 ± 1.50% of mCherry⁺ cells were GAD67⁺, and only 3.43 ± 1.22% were CaMKII⁺, confirming selective targeting of inhibitory neurons in GAD‑Cre mice (*n* = 6 slices/3 mice). **N**. Representative Western blot (left) and quantification (right) showing a significant increase of GluK1 protein expression in the *Grik1*^*OE*^ group compared to the mCherry control group (*n* = 3 mice, *p < 0.05, Na, K-ATPase was used as a loading control). **O**. After overexpressing GluK1 in the ACC of GAD-Cre mice, the mechanical withdrawal threshold in the von Frey test was significantly increased in the *Grik1*^*OE*^ SNI mice compared to the mCherry SNI mice, *n* = 7–8 mice, *p < 0.05 vs. shRNA(NC) SNI group, ^###^*p* < 0.001 vs. sham by two-tailed unpaired Student’s t-test. **P**. After overexpressing *Grik1* in the ACC of GAD-Cre mice, the withdrawal latency in the hot plate test of *Grik1*^*OE*^ SNI mice was significantly prolonged compared to the control SNI mice, *p < 0.05 vs. mCherry SNI group, ^##^*p* < 0.01 vs. mCherry sham group by two-tailed unpaired Student’s t-test. **R**. The percentage of time in open arms was significantly increased in *Grik1*^*OE*^ SNI mice, *p < 0.05 vs. mCherry SNI group by two-tailed unpaired Student’s t-test. **T**. The percentage of time in the central, the number of central entries, and the total distance in the OFT were significantly increased in the *Grik1*^*OE*^ SNI mice, *n* = 7–8 mice, *p < 0.05, **p < 0.01 vs. mCherry SNI group by two-tailed unpaired Student’s t-test

Given the upregulation of KAR function following chronic pain, we investigated whether cell-type-specific modulation of GluK1 could reverse neuropathic pain symptoms. We first utilized a viral genetic strategy to selectively knock down the GluK1 subunit in ACC glutamatergic neurons. We targeted CaMKIIa-Cre mice with AAV2/9-DIO-mCherry-miR30shRNA(*Grik1*)-WPRE (Fig. 8A). Immunofluorescence staining revealed that mCherry⁺ neurons were almost exclusively co-localized with the excitatory neuron marker CaMKII, with a co-localization rate of 98.57 ± 0.48%, confirming selective targeting of excitatory neurons in CaMKIIα‑Cre mice (Fig. 8B, C). Western blot analysis further confirmed that GluK1 protein expression was significantly reduced in the shRNA(*Grik1*) group compared to the shRNA(NC) control group (Fig. 8D). In sham mice, GluK1 knockdown had no effect on basal mechanical or thermal pain thresholds. In SNI mice, however, the mechanical withdrawal threshold was significantly increased in the shRNA(*Grik1*) group compared to the shRNA(NC) control (0.05 ± 0.01 g vs. 0.09 ± 0.02 g, *p < 0.05, Fig. 8E). Similarly, the withdrawal latency in the hot plate test was prolonged in the shRNA(*Grik1*) group (6.88 ± 0.12 s vs. 7.42 ± 1.56 s, *p < 0.05, Fig. 8F). Furthermore, we assessed anxiety-like behaviors using the elevated plus maze (EPM) and open field test (OFT) (Fig. 8G, I). In SNI mice, knockdown of GluK1 significantly reduced anxiety-like behavior in EPM compared with the shRNA(NC) group, whereas no significant difference was found in OFT (Fig. 8H, J). These results suggest that inactivation of GluK1 in ACC excitatory neurons results in both analgesic and partial anxiolytic effects.

Subsequently, we examined the therapeutic potential of targeting GluK1 in ACC GABAergic neurons. We induced the selective overexpression of GluK1 in GAD-Cre mice via AAV2/9-DIO-*Grik1*-3×FLAG-tWPA (Fig. 8K). Immunofluorescence staining showed that mCherry⁺ neurons predominantly co-localized with the inhibitory neuron marker GAD67 (70.45 ± 1.50%), confirming selective targeting of inhibitory neurons in GAD‑Cre mice (Fig. 8L, M). The proportion of mCherry⁺ cells not covered by GAD67 might be due to the technical limitations of the immunohistochemical detection itself, such as the sensitivity of the antibodies and the accessibility of the epitopes, as well as the fact that some transfected cells express a lower level of GAD67. Western blot analysis further demonstrated a significant increase in GluK1 protein expression in the *Grik1*^*OE*^ group compared to the mCherry control group (Fig. 8N). Overexpression of GluK1 did not alter basal nociceptive thresholds in sham mice. However, in SNI mice, the GluK1-overexpression (*GluK1*^*OE*^) group exhibited a significantly higher mechanical withdrawal threshold compared to the mCherry group (0.036 ± 0.007 g vs. 0.085 ± 0.017 g, *p < 0.05, Fig. 8O). Similarly, the hot plate withdrawal latency was prolonged in the *GluK1*^*OE*^ group (7.02 ± 0.11 s vs. 7.57 ± 0.18 s, *p < 0.05, Fig. 8P). Behavioral assessment in the EPM and OFT (Fig. 8Q, S) showed that SNI mice exhibited significantly reduced anxiety-like behaviors after GluK1 overexpression (Fig. 8R, T). These data collectively demonstrate that increased expression of GluK1 in ACC inhibitory neurons also produces analgesic and anxiolytic effects in neuropathic pain.

### Intra-ACC microinjection of the GluK1 agonist ATPA attenuates neuropathic and inflammatory pain

**Fig. 9:**
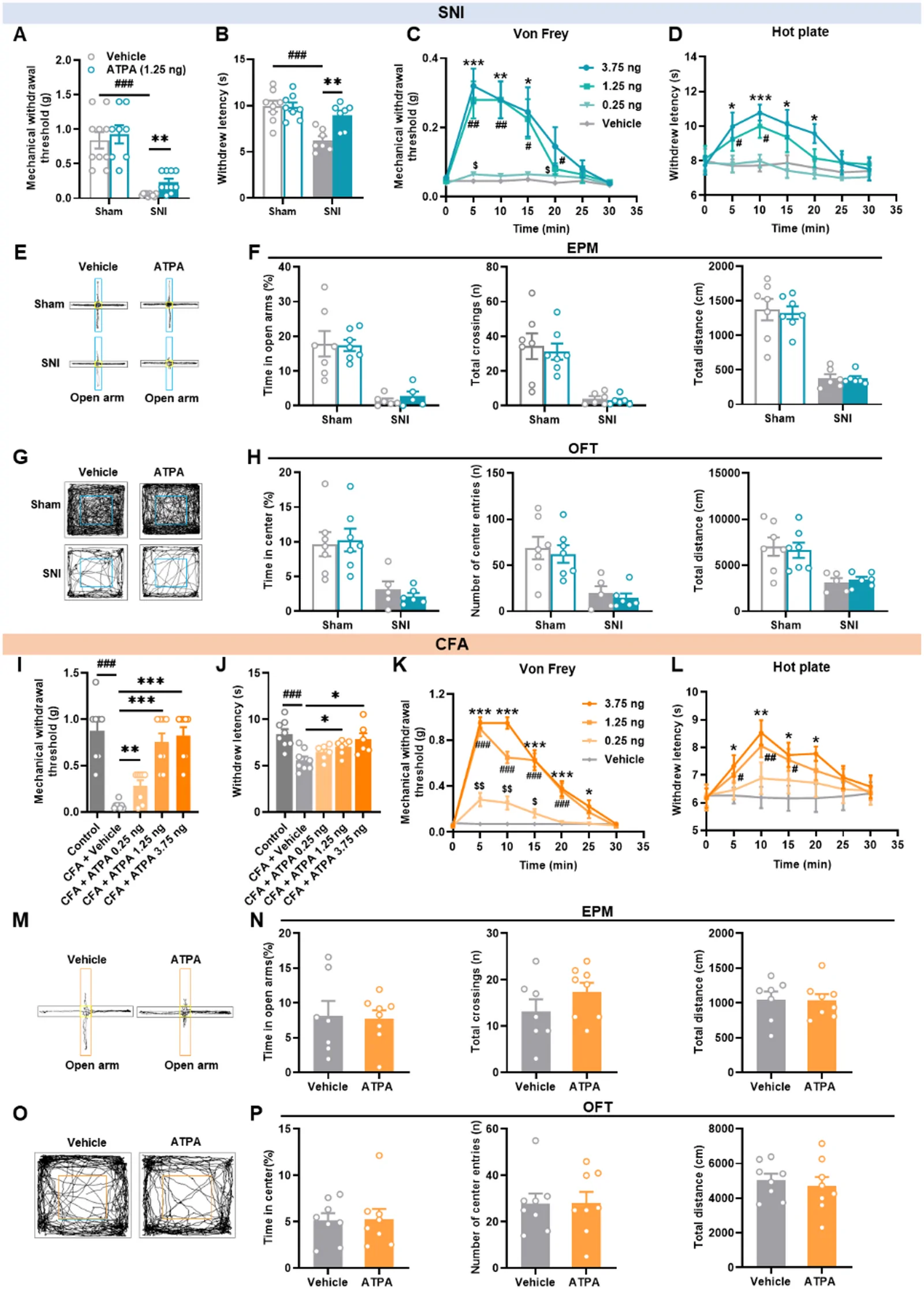
Microinjection of ATPA into ACC produces analgesia without anxiolytic effects. **A**. In the SNI model mice, after ACC microinjection of ATPA (1.25 ng), the mechanical withdrawal threshold (PWT) was increased, *n* = 7–9 mice, **p < 0.01 vs. saline SNI group, ^###^*p* < 0.001 vs. saline sham group by two-tailed unpaired Student’s t-test. **B**. In the SNI mice, after ACC microinjection of ATPA (1.25 ng), the thermal withdrawal latency was prolonged, *n* = 7–9 mice, **p < 0.01 vs. saline SNI group, ^###^*p* < 0.001 vs. saline sham group by two-tailed unpaired Student’s t-test. **C**, **D**. The time and dose curves showed the analgesic effect of ATPA (0.25, 1.25, 3.75 ng) in the von Frey test and the hot plate test in SNI mice, *n* = 7–8 mice, *p < 0.05, **p < 0.01, ***p < 0.001, ^#^*p* < 0.05, ^##^*p* < 0.01, ^#^*p* < 0.05 vs. saline, two-way ANOVA with Tukey post-hoc. **E**, **M**. Elevated plus maze (EPM) trajectory diagram. **F**. The administration of ATPA (1.25 ng) had no anxiolytic effect in the EPM in SNI mice, *n* = 7–8 mice. **G**, **O**. Open field test trajectory diagram. **H**. The administration of ATPA (1.25 ng) had no anxiolytic effect in the OFT in SNI mice, *n* = 7–8 mice. **I**. In the CFA model mice, intra-ACC injection of ATPA (0.25, 1.25, 3.75 ng) produced an analgesic effect in a dose-dependent manner in the von Frey test. *n* = 8–10 mice, ^##^*p* < 0.01 vs. sham by two-tailed unpaired Student’s t-test, **p < 0.01, ***p < 0.001 vs. CFA, one-way ANOVA with Tukey post-hoc. **J**. In the hot plate test, intra-ACC injection of ATPA (0.25, 1.25, 3.75 ng) produced an analgesic effect in a dose-dependent manner in CFA model mice. *n* = 8–10 mice, ^##^*p* < 0.01 vs. sham by two-tailed unpaired Student’s t-test, *p < 0.05 vs. CFA, one-way ANOVA with Tukey post-hoc. **K**-**L**. The time and dose curves showed the analgesic effect of ATPA in the von Frey test and the hot plate test in CFA mice, *n* = 8–10 mice, *p < 0.05, **p < 0.01,***p < 0.001, ^#^*p* < 0.05, ^##^*p* < 0.01, ^###^*p* < 0.001, ^#^*p* < 0.05, ^##^*p* < 0.01 vs. saline, two-way ANOVA with Tukey post-hoc. **N**. The administration of ATPA (1.25 ng) had no anxiolytic effect in the EPM in CFA mice (*n* = 8–10 mice).**P**. The administration of ATPA (1.25 ng) had no anxiolytic effect in the OFT in CFA mice (*n* = 8–10 mice)

Next, we investigated the therapeutic potential of pharmacological KAR modulation. We assessed whether microinjection of the GluK1 agonist ATPA into the ACC exerts analgesic effects in neuropathic pain. In the SNI model after 7 days, bilateral intra-ACC administration of ATPA (1.25 ng) significantly attenuated both mechanical allodynia and thermal hyperalgesia (Von-Frey: 0.24 ± 0.047 g vs. 0.05 ± 0.007 g in the saline group, **p < 0.01; hot-plate: 8.96 ± 0.51 s vs. 6.25 ± 0.44 s in the saline group, **p < 0.01; Fig. 9A, B). The assessment of dose-response and temporal profiles indicated that ATPA (1.25 and 3.75 ng) markedly elevated pain thresholds within 5–10 min following delivery, with this effect enduring for roughly 30 min (Fig. 9C, D). The data indicate that ATPA produces a fast but temporary analgesic effect. Simultaneously, we assessed the influence of ATPA on comorbid anxiety-like behaviours that manifested concurrently following 2 weeks of SNI. SNI mice exhibited a marked decrease in open-arm duration (EPM) and central zone exploration (OFT), but intra-ACC treatment of ATPA (1.25 ng) did not alleviate these anxiety-like behaviours (Fig. 9E–H). The results indicate a functional dissociation, whereby the analgesic effects of ATPA function independently of the pathways regulating anxiety.

To validate these findings in an inflammatory pain context, we assessed the analgesic efficacy of ATPA after 3 days of the Complete Freund’s Adjuvant (CFA) model. Following intra-ACC injection of ATPA (0.25, 1.25, and 3.75 ng), CFA-induced mice exhibited a dose-dependent increase in mechanical paw withdrawal thresholds and thermal withdrawal latencies (Fig. 9I–L). Mirroring the kinetic profile observed in neuropathic pain, the analgesic effect in inflammatory pain peaked within 5–10 min and lasted approximately 30 min. However, consistent with the SNI results, ATPA treatment did not alter anxiety-like behaviors in 2 weeks of CFA mice (Fig. 9M–P). In summary, ATPA produces dose- and time‑dependent analgesia in both neuropathic and inflammatory pain models but does not exhibit anxiolytic effects. We hypothesize that ATPA‑induced analgesia is mediated by activation of KARs on GABAergic neurons, whereas the absence of anxiolytic action suggests that anxiety‑related behaviors in the ACC are likely regulated by glutamatergic circuits engaged by ATPA. However, these interpretations remain speculative and require direct experimental validation in future studies.

## Discussion

**Fig. 10:**
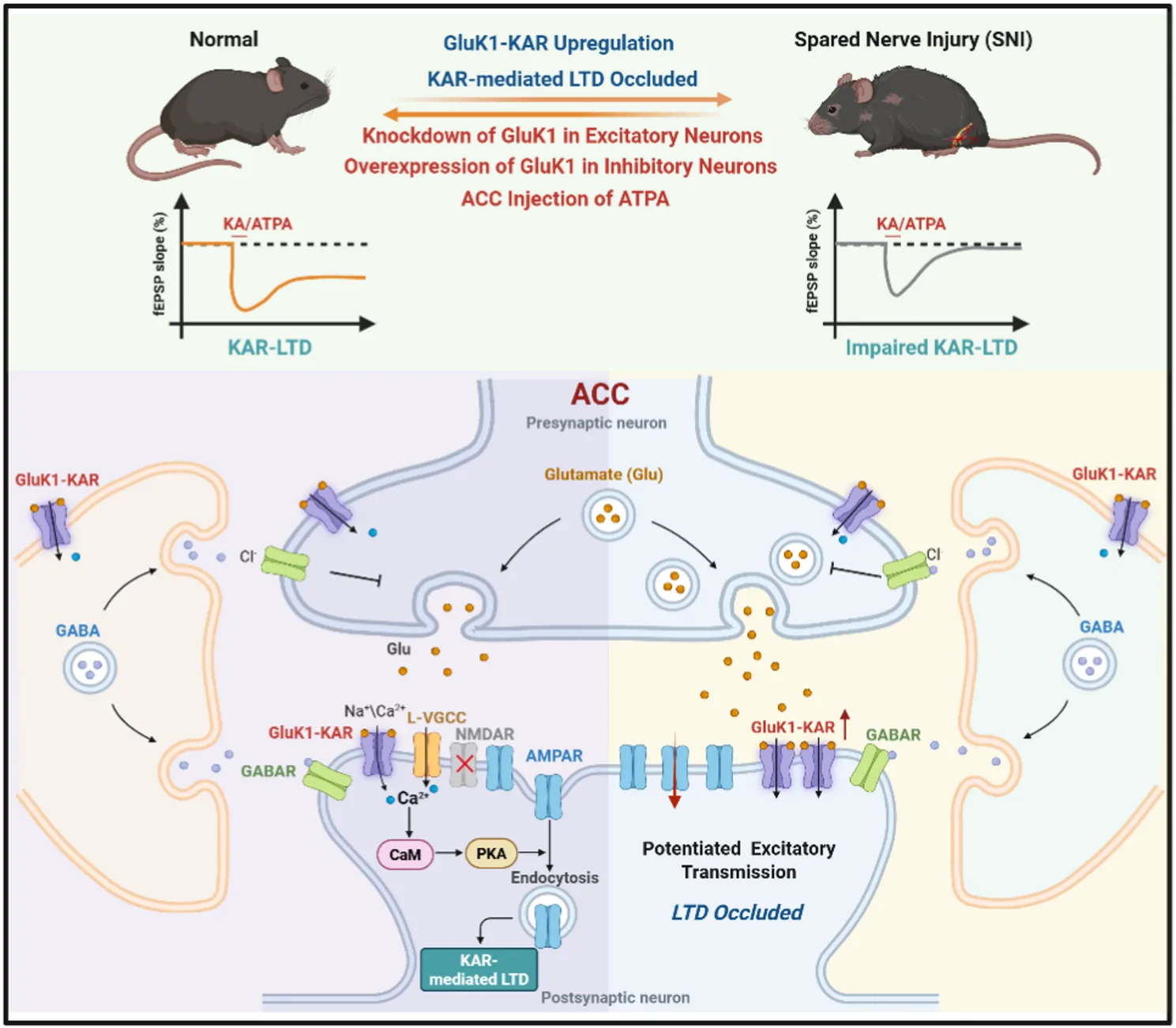
The mechanism model of KAR-mediated LTD in the ACC involvement in chronic pain regulation. KAR-mediated LTD that operates via GluK1-containing KARs, L-type voltage-gated calcium channels (L-VGCCs), and the Ca^2+^-activated PKA signaling pathway, independent of canonical NMDAR mechanisms. Presynaptic inhibition via GABA_A_ receptors was also involved in KAR-mediated LTD. KAR-mediated LTD is functionally impaired in mice in a chronic pain model, despite upregulation of GluK1 subunit expression and increased KAR-mediated EPSCs. Knocking down GluK1 in excitatory neurons or overexpressing it in inhibitory neurons is an effective therapy for chronic pain. GluK1-containing KARs in the ACC represent a potential intervention node for chronic pain

LTD is one type of synaptic plasticity in the CNS, which is related to the formation and development of chronic pain [1, 36]. Although KARs are recognised mediators of LTP in the ACC, the characteristics and functional roles of GluK1-containing KAR-mediated LTD in this region remain poorly characterized. In the current study, using a combinatorial approach involving multichannel field potential recording, whole-cell patch-clamp recording, pharmacology, and behavioral assays, we identified a novel form of KAR-dependent LTD in the ACC contributing to chronic pain. This LTD is modulated by L-type voltage-gated calcium channels (L-VGCCs) and the PKA signaling pathway. Furthermore, the inhibitory synaptic transmission involved in KAR-mediated LTD is demonstrated by its blockade via the GABA_A_ receptor antagonist, picrotoxin. In mice with SNI, KAR-mediated LTD was completely occluded, despite an upregulation of GluK1 subunit expression and increased KAR-mediated EPSCs (Fig. 10). Additionally, the inhibitory effect of KA on excitatory synapses was attenuated in SNI mice relative to sham controls. Behaviorally, specific knockdown of GluK1 in excitatory neurons or its overexpression in inhibitory neurons elicited analgesia in SNI mice. Similarly, intra-ACC microinjection of ATPA dose-dependently reversed the decreasing mechanical and thermal withdrawal thresholds in both neuropathic and inflammatory pain models but did not affect the anxiety behaviors. Collectively, our results suggest that aberrant KAR-mediated LTD in the ACC is involved in the maintenance of chronic pain. KARs may act at multiple targets to affect ACC. Similar findings have been reported in the spinal cord synapses [16, 25].

Further research is required to explore the potential function of KARs in pre- versus post-synaptic and excitatory versus inhibitory synapses in the ACC.

### KAR-mediated LTD in the ACC

LTD has conventionally been ascribed to the activation of NMDA receptors or metabotropic glutamate receptors [4, 9]. Nonetheless, growing data suggests that KARs can autonomously induce a unique variant of LTD. Sustained activation of postsynaptic kainate receptors (KARs) in the hippocampus induces the endocytosis of AMPA receptors, a mechanism that necessitates the ionotropic activity of KARs, irrespective of N-methyl-D-aspartate receptor (NMDAR) or metabotropic glutamate receptor (mGluR) signalling [19]. In the present study, we characterized a novel form of KAR-mediated LTD within the ACC. Using MED64 multielectrode array recordings, we observed that perfusion of the KAR agonist kainic acid (KA) induced LTD in a dose-dependent manner. This chemically induced plasticity exhibited a rapid onset and persisted for over three hours. Network analysis revealed that the KA application increased the number of channels displaying synaptic depression, thereby reducing the total area of activated synapses. Similarly, the GluK1-selective agonist ATPA elicited dose-dependent LTD, which was abolished by the GluK1 antagonist UBP310. Furthermore, whole-cell patch-clamp recordings demonstrated that KA-induced LTD persists even in the presence of the AMPA receptor antagonist GYKI 53,655. These findings are consistent with prior reports of exclusive KAR-mediated LTD in the hippocampus and perirhinal cortex [17–19]. Crucially, we determined that this chemically induced KAR-mediated LTD operates independently of conventional low-frequency stimulation (LFS)-induced LTD; both forms can coexist at ACC synapses. These results suggest that AMPAR-mediated LTD and KAR-mediated LTD represent parallel plasticity mechanisms in the ACC. Future investigations will need to explore whether specific electrical stimulation protocols can physiologically recruit this KAR-mediated LTD and whether chemical-induced KAR-mediated LTD and LFS-induced LTD are genuinely separate.

### The Ca^2+^-activated PKA signaling pathway is required for the KAR-mediated LTD

By analogy with the hippocampus, two major forms of LTD have been observed in the ACC synapses, including NMDAR-LTD and mGluR-LTD. Both forms of LTD require postsynaptic modification of AMPARs for their expression. Specifically, NMDAR-LTD depends on Ca^2+^ influx through NMDARs to recruit Calmodulin (CaM) and activate protein phosphatase 1 (PP1) for dephosphorylation of AMPARs and depression of synaptic responses. On the other hand, mGluR-LTD is mediated by mGluR1 and L-VGCCs, under the control of PKC-dependent metaplasticity [1, 2]. In the present study, we demonstrated that the induction of KAR-mediated LTD by kainic acid or ATPA in the ACC is strictly dependent on the activation of GluK1-containing KARs. We further identified the critical postsynaptic signaling cascades involved. Pharmacological inhibition of L-VGCCs abolished KAR-mediated LTD, whereas antagonists targeting NMDARs or calcium-permeable AMPARs (CP-AMPARs) had no effect. Furthermore, the protein kinase A (PKA) inhibitor KT5720, but not the PKC inhibitor chelerythrine chloride, prevented KAR-mediated LTD induction. These results suggest that a Ca^2+^-activated PKA signaling pathway mediates KAR-induced AMPAR endocytosis, in agreement with observations at hippocampal CA1 synapses [19]. In the perirhinal cortex, LTD of KAR-mediated transmission (EPSC_KA_ LTD) proceeds via a distinct mechanism compared with AMPAR-LTD: while it requires postsynaptic Ca^2+^ elevation, it operates independently of NMDARs or L-VGCCs, instead relying on tonic mGluR5 modulation and PKC-PICK1 PDZ domain interactions [17]. The diversity of these molecular mechanisms of KAR-related LTD highlights the complex role of KARs in LTD regulation. We propose that, contingent upon the activation pattern (transient vs. sustained), KARs modulate synaptic AMPARs and plasticity via distinct signaling pathways.

### GABA receptor-mediated presynaptic inhibition is involved in KAR-mediated LTD

Previous studies have established that KARs are distributed both pre- and postsynaptically, regulating the release of excitatory or inhibitory neurotransmitters in regions such as the dorsal horn, basolateral amygdala, and ACC [24, 25, 37]. Given the suppressive nature of KAR-mediated LTD, we investigated whether inhibitory transmission mediated by GABA receptors contributes to its induction. We observed that GABA_A_ receptor antagonists abolished KAR-mediated LTD induced by either kainic acid or ATPA. Notably, the effect of KAR activation is temporally dependent: while transient activation (via bath application) potentiates the release of both excitatory and inhibitory neurotransmitters, sustained activation suppresses both forms of transmission. Furthermore, whole-cell patch-clamp recordings revealed that kainic acid treatment increased the paired-pulse ratio (PPR) during LTD induction, implicating a presynaptic inhibition participation. This data is consistent with the finding that the mGluR-LTD also involves presynaptic inhibition in CA1 of the hippocampus [38]. It should be noted that our evidence for GABAergic involvement is primarily based on picrotoxin blockade and paired‑pulse ratio changes, supporting a role for GABA_A_ receptor-mediated presynaptic inhibition in KAR-mediated LTD induction. However, these data do not identify the specific inhibitory circuit elements involved. Definitive elucidation of the underlying circuit mechanism will require direct recordings from identified interneurons or miniature IPSC analysis during KA-induced LTD. In the ACC, GluK1 subunits are selectively expressed in interneurons; their activation elicits robust depolarization, thereby driving synaptic GABA release and enhancing tonic GABA currents. Thus, KARs can bidirectionally modulate synaptic transmission [15]. This complex regulation suggests that KAR modulation of network excitability may indirectly influence GABAergic inhibition, thereby disturbing the critical excitatory–inhibitory (E/I) balance in the ACC. More detailed studies are needed to elucidate the role of GABA receptors in the KAR-mediated LTD.

### The function of ACC KAR-mediated LTD in chronic pain

The ACC functions as a crucial center for processing nociception and related emotional states in the mammalian brain. The hyperactivation of the ACC is closely associated with chronic pain syndromes and their accompanying emotional states, including fear, anxiety, and depression [1, 33, 39–41]. LTD in the ACC is significantly altered after peripheral tissue injury. In rodent models of digit or tail amputation, the induction of LTD in the ACC is significantly impaired [4, 9]. Nerve injury downregulates caspase-3 levels, which consequently disrupts AMPAR endocytosis and obstructs LTD induction [5]. In accordance with these findings, our data indicate that KAR-mediated LTD is effectively blocked in the ACC of SNI model mice, accompanied by a markedly reduced induction rate in multi-channel field potential recordings. The KAR-mediated LTD loss occurs alongside an upregulation of GluK1-containing KARs and an increase in KAR-mediated excitatory synaptic transmission. Moreover, the treatment of kainic acid diminishes the excitation/inhibition (E/I) ratio in sham-operated mice, but this effect is nullified in SNI mice. This indicates that kainic acid’s inhibitory effect on excitatory synaptic transmission is less pronounced in nerve-injured mice compared to sham mice. Based on these observations, we propose that the suppression of KAR-mediated LTD constitutes a key pathological feature of chronic pain. This functional loss likely fosters network hyperexcitability within pain circuits, thereby contributing to the maintenance of the chronic pain state. Future study is required to investigate the role of restoring KAR-mediated LTD in chronic pain.

### The possibility of treating chronic pain with targeted KARs therapy

KARs are widely expressed along the pain neuraxis, including the dorsal root ganglion (DRG), spinal cord, ACC, and insular cortex, where they are essential for mediating fast synaptic transmission and plasticity [14, 25, 37]. Prior research indicates that KARs, particularly those containing the GluK1 subunit, are integral to chronic pain pathology [27, 42]. In the present study, we observed that SNI-induced chronic pain is associated with potentiated KAR-mediated synaptic transmission and upregulated KAR expression in the ACC. Targeted knockdown of GluK1-containing KARs in excitatory neurons, or their overexpression in inhibitory neurons, elicited both analgesic and anxiolytic effects in SNI mice. Furthermore, we found that intra-ACC microinjection of the GluK1 agonist ATPA dose-dependently attenuated nerve injury- and inflammation-induced mechanical and thermal hyperalgesia, without altering anxiety levels. Our data corroborates the discovery that intrathecal administration of ATPA has an analgesic effect within the spinal cord [43]. The differential effects of viral manipulation and ATPA on anxiety-like behaviours may indicate distinct temporal and circuit-level actions—chronic genetic modulation versus acute pharmacological stimulation—though this interpretation necessitates direct electrophysiological validation in future studies and should currently be regarded as hypothetical. It is important to recognise that our present behavioural investigations should be viewed as indicative of a functional role for cell-type-specific GluK1 regulation, rather than as conclusive evidence that KAR-mediated LTD restoration or E/I balance normalisation is the causal mechanism for the observed analgesia. Future electrophysiological investigations are necessary to directly associate behavioural effects with alterations in synaptic plasticity or network excitability. Nevertheless, some preclinical studies have demonstrated that GluK1-selective antagonists effectively alleviate pain behaviors in diverse models. The antagonists (e.g., LY293558) have shown efficacy in clinical trials for inflammatory, postoperative, and migraine pain, with an acceptable safety profile [15, 27]. These previous data and our findings suggest the complexity of KAR’s function in the CNS [44]. Collectively, a simple drug targeted at KARs may be difficult, and a mixed-action drug on KARs, especially different subtypes, may be useful for future drug development for treating pain and anxiety. In addition to direct agonism or antagonism, altering downstream signalling pathways to repair impaired LTD offers a viable alternative approach for future analgesic development.

## Conclusion

This study found a unique kind of KAR-dependent LTD in the ACC associated with chronic pain. The KAR-mediated LTD required activation of GluK1-containing KARs, L-type voltage-gated calcium channels (L-VGCCs), and the Ca^2+^-activated PKA signaling pathway, independent of traditional NMDAR processes. We identify the involvement of GABA_A_ receptor-mediated presynaptic inhibition, highlighting a complex regulation of the excitation/inhibition (E/I) balance. This KAR-mediated LTD is implicated in the pathogenesis of chronic pain. In mice with SNI, KAR-mediated LTD was impaired, despite an upregulation of GluK1 subunit expression and increased KAR-mediated EPSCs (Fig. 10). Furthermore, by employing cell-type-specific viral strategies, the downregulation of GluK1 in excitatory neurones or its overexpression in inhibitory neurones produces significant analgesic effects. Moreover, intra-ACC microinjection of the GluK1-selective agonist ATPA significantly attenuates mechanical and thermal hyperalgesia in both neuropathic and inflammatory pain models but does not affect the anxiety behaviors. Collectively, our findings indicate that GluK1-containing KAR-mediated LTD in the ACC is linked to the persistence of chronic pain. Pharmacological activation of GluK1 or cell-type-specific modulation of GluK1 expression produces analgesic effects, suggesting that GluK1-containing KARs may serve as a candidate intervention node for chronic pain. However, the causal link between LTD restoration and analgesia has not been directly established, and the translational relevance of these findings requires further preclinical validation. A limitation of the current investigation is the exclusive use of adult male mice. Considering the recognised sexual dimorphisms in pain processing and synaptic plasticity, subsequent research should incorporate both sexes to ascertain whether the identified KAR-mediated LTD and its analgesic properties exhibit sex dependence.

## Data Availability

The datasets used and/or analysed during the current study are available from the corresponding author on reasonable request.

## Abbreviations

ACC: Anterior cingulate cortex
AMPARs: 2-amino-3-(3-hydroxy-5-methyl-isoxazol-4-yl) propanoic acid receptors
ATPA: (RS)-2-amino-3-(3-hydroxy-5-tert-butylisoxazol-4-yl)propanoic acid
CaM: Calmodulin
CFA: Complete Freund’s adjuvant
CNS: Central nervous system
CREB: cAMP response element-binding protein (CREB)
EPSCs: Excitatory postsynaptic currents
EPSPs: Excitatory postsynaptic potentials
GluK1: Kainate receptor K1 subunit
KAR: Kainate receptor
LFS: Low-frequency stimulation
LTD: Long-term depression
LTP: Long-term potentiation
NMDARs: N-methyl-D-aspartate receptors
PKA: Protein kinase A
PKC: Protein kinase C
TBS: Theta burst stimulation
SNI: Spared nerve injury
